# Momentary Influences on Self-Regulation in Two Populations With Health Risk Behaviors: Adults Who Smoke and Adults Who Are Overweight and Have Binge-Eating Disorder

**DOI:** 10.3389/fdgth.2022.798895

**Published:** 2022-03-18

**Authors:** Emily A. Scherer, Stephen A. Metcalf, Cady L. Whicker, Sophia M. Bartels, Michael Grabinski, Sunny Jung Kim, Mary Ann Sweeney, Shea M. Lemley, Hannah Lavoie, Haiyi Xie, Patrick G. Bissett, Jesse Dallery, Michaela Kiernan, Michael R. Lowe, Lisa Onken, Judith J. Prochaska, Luke E. Stoeckel, Russell A. Poldrack, David P. MacKinnon, Lisa A. Marsch

**Affiliations:** ^1^Center for Technology and Behavioral Health, Geisel School of Medicine at Dartmouth College, Lebanon, NH, United States; ^2^Department of Public Health and Primary Care, University of Cambridge, Cambridge, United Kingdom; ^3^Department of Health Behavior, Gillings School of Global Public Health, University of North Carolina, Chapel Hill, NC, United States; ^4^Department of Health Behavior and Policy, School of Medicine, Virginia Commonwealth University, Richmond, VA, United States; ^5^Massey Cancer Center, Virginia Commonwealth University, Richmond, VA, United States; ^6^Department of Health Education and Behavior, University of Florida, Gainesville, FL, United States; ^7^Department of Psychology, Stanford University, Stanford, CA, United States; ^8^Department of Psychology, University of Florida, Gainesville, FL, United States; ^9^Stanford Prevention Research Center, Department of Medicine, Stanford University, Stanford, CA, United States; ^10^Department of Psychological and Brain Sciences, Drexel University, Philadelphia, PA, United States; ^11^National Institute on Aging, National Institutes of Health, Bethesda, MD, United States; ^12^Department of Psychology, Arizona State University, Tempe, AZ, United States

**Keywords:** momentary self-regulation, ecological momentary assessment (EMA), smoking, binge-eating disorder, overweight, obesity, behavioral health, digital health

## Abstract

**Introduction:**

Self-regulation has been implicated in health risk behaviors and is a target of many health behavior interventions. Despite most prior research focusing on self-regulation as an individual-level trait, we hypothesize that self-regulation is a time-varying mechanism of health and risk behavior that may be influenced by momentary contexts to a substantial degree. Because most health behaviors (e.g., eating, drinking, smoking) occur in the context of everyday activities, digital technologies may help us better understand and influence these behaviors in real time. Using a momentary self-regulation measure, the current study (which was part of a larger multi-year research project on the science of behavior change) used ecological momentary assessment (EMA) to assess if self-regulation can be engaged and manipulated on a momentary basis in naturalistic, non-laboratory settings.

**Methods:**

This one-arm, open-label exploratory study prospectively collected momentary data for 14 days from 104 participants who smoked regularly and 81 participants who were overweight and had binge-eating disorder. Four times per day, participants were queried about momentary self-regulation, emotional state, and social and environmental context; recent smoking and exposure to smoking cues (smoking sample only); and recent eating, binge eating, and exposure to binge-eating cues (binge-eating sample only). This study used a novel, momentary self-regulation measure comprised of four subscales: momentary perseverance, momentary sensation seeking, momentary self-judgment, and momentary mindfulness. Participants were also instructed to engage with Laddr, a mobile application that provides evidence-based health behavior change tools *via* an integrated platform. The association between momentary context and momentary self-regulation was explored *via* mixed-effects models. Exploratory assessments of whether recent Laddr use (defined as use within 12 h of momentary responses) modified the association between momentary context and momentary self-regulation were performed *via* mixed-effects models.

**Results:**

Participants (mean age 35.2; 78% female) in the smoking and binge-eating samples contributed a total of 3,233 and 3,481 momentary questionnaires, respectively. Momentary self-regulation subscales were associated with several momentary contexts, in the combined as well as smoking and binge-eating samples. For example, in the combined sample momentary perseverance was associated with location, positively associated with positive affect, and negatively associated with negative affect, stress, and tiredness. In the smoking sample, momentary perseverance was positively associated with momentary difficulty in accessing cigarettes, caffeine intake, and momentary restraint in smoking, and negatively associated with temptation and urge to smoke. In the binge-eating sample, momentary perseverance was positively associated with difficulty in accessing food and restraint in eating, and negatively associated with urge to binge eat. While recent Laddr use was not associated directly with momentary self-regulation subscales, it did modify several of the contextual associations, including challenging contexts.

**Conclusions:**

Overall, this study provides preliminary evidence that momentary self-regulation may vary in response to differing momentary contexts in samples from two exemplar populations with risk behaviors. In addition, the Laddr application may modify some of these relationships. These findings demonstrate the possibility of measuring momentary self-regulation in a trans-diagnostic way and assessing the effects of momentary, mobile interventions in context. Health behavior change interventions may consider measuring and targeting momentary self-regulation in addition to trait-level self-regulation to better understand and improve health risk behaviors. This work will be used to inform a later stage of research focused on assessing the transdiagnostic mediating effect of momentary self-regulation on medical regimen adherence and health outcomes.

**Clinical Trial Registration:**

ClinicalTrials.gov, Identifier: NCT03352713.

## Introduction

Self-regulation refers to a person's ability to manage cognitive, motivational, and emotional resources to act in accordance with their long-term goals (1). Self-regulation has been implicated in many health risk behaviors, such as poor diet, physical inactivity, gambling, and use of tobacco and other substance use (2–6). Modifying self-regulation has therefore been a target of many health behavior interventions (7, 8). The literature investigating the role of self-regulation in health risk behaviors, and the interventions targeting self-regulation, have typically focused at the individual trait level, rather than viewing it as a process that may vary moment to moment within an individual in response to contextual situations and external and internal stimuli (9). While a limited number of studies have evaluated self regulation on a momentary level (10), these studies have not employed a momentary self-regulation scale and review of the literature has identified the need for further research at the moment level to understand self regulation as a mechanism of behavior change (11).

We hypothesize that although individuals may vary in their mean level of self-regulation, momentary contexts may influence (increase or decrease) self-regulation to a substantial degree, making self-regulation a time-varying construct. This hypothesis is part of a broader framework examining self-regulation as a putative target of behavior change interventions. Roos and Witkiewitz (9), developed a framework of self-regulation in which an individual's self-regulation is a mechanism of behavior change, and is considered both as an individual trait, and as a time-varying construct which is influenced by both internal and external factors. The hypothesis that self-regulation varies in the moment and in response to internal and external contexts is also consistent with Gross' extended process model of emotion regulation (12), and other applied research on emotion regulation (13–15). If true, explorations of the role of self-regulation, and interventions focused on modifying self-regulation, should consider (and measure) momentary variation in self-regulation in addition to trait-level self-regulation.

Advances in digital technologies have created unprecedented opportunities to assess and modify self-regulation and health behavior on a moment-by-moment basis (16, 17). Because most health behaviors (e.g., eating, drinking, and smoking) occur in the context of everyday activities, digital technologies may help us better understand and influence these behaviors in real time (18). For example, mobile technologies enable ecological momentary assessment (EMA), a methodology that prompts individuals to respond to specific queries on mobile devices (19). EMA enables real-time monitoring of individuals' behavior while they engage in everyday activities over time (20). The frequent, longitudinal assessment afforded by EMA in naturalistic contexts may clarify the roles of mechanisms, or reveal new mechanisms, in changing self-regulatory behavior.

The current study used EMA to assess the time-varying nature of self-regulation and examine whether self-regulation changes—increases and decreases—on a momentary basis in naturalistic, non-laboratory settings. This study offers the opportunity to test hypotheses regarding self-regulation's time-varying nature while using a scale of momentary self-regulation. This work is part of a broader exploration of the ontology of self-regulation supported by the National Institutes of Health's Science of Behavior Change (SOBC) initiative (1). The goal of this SOBC initiative is to make behavior change research more targeted and systematic by employing an “experimental medicine” approach to understanding common mechanism underlying behavior change (21). Consistent with the goal of understanding processes that underly multiple health risk behaviors, we assessed if self-regulation (as a putative target of behavior change) can be engaged and manipulated on a momentary basis in naturalistic, non-laboratory settings among samples from two exemplar populations with health risk behaviors: individuals who smoke and individuals who are overweight and have binge-eating disorder, each of whom were asked to respond to EMA queries on mobile devices four times per day for 2 weeks. There were no specific hypotheses about how each momentary self-regulation subscale would differ by context or which contexts would affect which self-regulation subscale. Instead, this exploratory study was aimed at providing preliminary evidence that momentary self-regulation may vary in response to different environments throughout everyday life. Similarly, while both populations engage in health risk behaviors, the etiology of each health risk behavior likely differs, with smoking characterized by an addiction to nicotine (22), whereas the etiology of binge eating, including potential relations with addiction (23, 24), is less well understood (25). While we might also expect to see differences between the samples in whether and which contexts affect momentary self-regulation, no specific sample differences were hypothesized given the novelty of the momentary self-regulation measurement.

The goals of these exploratory analyses of the EMA data were (1) to examine whether momentary self-regulation changed in different contexts (that may increase or decrease self-regulation) and in response to the Laddr® digital self-regulation application (26), and (2) to explore whether use of the digital self-regulation intervention modified the relationship between contexts and momentary self-regulation.

This work will also be used to inform a later stage of research focused on assessing the transdiagnostic mediating effect of momentary self-regulation on medical regimen adherence and health outcomse among these same two exemplar populations with risk behaviors.

## Materials and Methods

### Study Design

This was a one-arm, open-label exploratory study with prospective momentary data collection. Although Laddr was employed in the current study, it was not used as an intent-to-treat intervention to promote health behavior change. Rather, to achieve the scientific aims of this study, Laddr enabled EMA data collection and provided access to therapeutic content that may increase self-regulation.

The target sample size was 50 participants who smoked and 50 participants who were overweight and had binge-eating disorder. An additional set of participants (target of 25 participants from each population) was recruited to participate in passive-sensing mobile data collection *via* wrist sensors in addition to the study intervention and data collection (The passive-sensing data results will be reported elsewhere). Because the additional participants received the same mobile intervention and provided data through the same baseline and momentary questionnaires, these participants are included in the study summary that follows. The decision to include the passive-sensing study participants occurred prior to any analyses.

The target sample size of 50 participants per population was determined to provide an estimated 80% power to detect a difference between contexts in mean momentary self-regulation that is 0.2–0.3 times a standard deviation (SD). This assumed completion of at least two assessments per day for 2 weeks at an 80% signal response rate, with intraclass correlations ranging from 0.001 to 0.3, and prevalence of a context of interest of 10% and 30% of responses. The addition of the 25 passive-sensing study participants in each sample provided increased power beyond these estimates.

### Participant Eligibility

The larger project of which the present study is part also includes a study that used functional magnetic resonance imaging (fMRI) in addition to self-report of self-regulation (1). While the participants in the present study were not enrolled in the fMRI study, the eligibility criteria were harmonized between the two studies in order to have comparable samples for future comparison of behavioral and fMRI results. Thus, some of the eligibility criteria for the current study were influenced by eligibility requirements for the fMRI study [e.g., upper limit for body mass index (BMI) was influenced by fMRI capacity].

All participants were required to be aged 21–50 years, sufficiently proficient in understanding English to provide informed consent, and able to use a smartphone. Exclusion criteria were: any current (non-nicotine) substance use disorder evaluated based on self-report (participants were not excluded based on use of substances), being pregnant or having plans to become pregnant in the next 3 months, self-reported lifetime history of a major psychiatric disorder (e.g., schizophrenia and bipolar disorder but not major depressive disorder), current use of any medication for psychiatric reasons (e.g., stimulants, mood stabilizers, or antidepressants), current use of prescription pain medication, current use of any smoking cessation medication (participants were not excluded based on use of short-acting nicotine replacement therapy), current use of weight-loss medication or prior weight-loss surgery, current nighttime shift work, and current obstructive sleep apnea.

#### Samples Including Passive Mobile Data Collection

In addition to the criteria above, participants who additionally engaged in the passive mobile-sensing portion of the study were required to be able to use a study-provided smartphone, to be comfortable wearing a wrist sensor on each wrist, and to travel to the research center at the beginning and end of the 14-day study period.

#### Smoking Sample

In addition to the criteria above, participants in the smoking sample must have smoked 5 or more tobacco cigarettes per day for the past year. They could not be overweight or obese; their BMI must have been ≥17 and <27 kilograms per square meter (kg/m^2^). Additionally, participants who smoked could not engage in binge-eating behavior as determined by a subset of questions (#8–10), on the Questionnaire on Eating and Weight Patterns-5 (QEWP-5; (27). A current desire to quit smoking was not included in the eligibility criteria as this was not an intervention study for smoking cessation.

#### Binge-Eating Sample

In addition to the criteria above, participants in the binge-eating sample had a BMI between 27 and 45 kg/m^2^, inclusive, and had binge-eating disorder according to the Diagnostic and Statistical Manual of Mental Disorders, Fifth Edition (DSM-5) criteria (28). They were required to be non-smoking, defined as having no cigarettes in the past 12 months. Additionally, they could not exhibit any compensatory behavior (e.g., purging, excessive exercise, or fasting). They could not have lost more than 10 pounds in the past 6 months, and they could not be enrolled in an in-person weight-loss program. They also could not be on a special diet for a serious health condition.

### Recruitment Procedure

Study advertisements were posted online (e.g., Craigslist, Facebook) as well as in physical locations in the Dartmouth College community around New Hampshire and Vermont, including in health centers and non-healthcare locations in the community (e.g., convenience stores, bus stops). Participants in the target samples of 50 without passive sensing were recruited nationally throughout the United States, including districts and territories. Participants in the target samples of 25 who additionally participated in passive mobile sensing were recruited locally in the region around Dartmouth College. Interested individuals were directed to an eligibility screening questionnaire on Dartmouth's Research Electronic Data Capture (REDCap) online platform (29, 30). Recruitment and data collection occurred from January through December 2018. Recruitment for the national smoking and binge-eating samples continued until 50 participants from each population had completed at least 10% of their momentary assessment questionnaires. Recruitment for the mobile-sensing smoking and binge-eating samples continued until 25 participants from each population had completed at least 10% of their momentary assessment questionnaires. Although, recruitment goals were based on participants completing at least 10% of the momentary assessments, completing fewer than 10% of the momentary assessments was not an exclusion criterion.

### Study Conduct

Individuals who met the eligibility criteria for the study based on responses to the online screening tool were scheduled for a 14-consecutive-day study period. The subset of participants who also participated in the passive-sensing mobile data collection portion of the study (not described here) were required to make in-person visits at the beginning and end of the study period. All participants provided informed consent, and the study was approved by the Dartmouth College institutional review board.

All participants were asked to use the Laddr mobile intervention daily during the 14-day study period. Informed by over 20 years of NIH-funded research and dozens of randomized trials (31–35), Laddr offers evidence-based health behavior change tools *via* an integrated platform to target a wide range of behavioral problems, including smoking, binge eating, alcohol use, other substance use, depression, panic, and anxiety. Each section of Laddr provides behavior-specific tools for activating behavior change, solving problems, overcoming obstacles to effective behavior change, and developing skills for maintenance of behavior change. Participants were asked to use Laddr to learn information about smoking and binge eating, as applicable, and to create personalized goals regarding their health risk behavior. Participants were instructed to follow the guides, where they could learn about their cues to smoking and binge eating and then practice healthier alternatives when faced with their cues, such as engaging in breathing exercises or going for a walk. Participants were asked to use Laddr to track progress toward their goals throughout the study period. In the present study, the samples who smoked and who had binge-eating disorder were encouraged to use the smoking and binge-eating disorder sections of Laddr, respectively.

At baseline, participants completed a battery of self-regulation questionnaires (17 questionnaires) as well as questionnaires about demographic information and smoking or binge-eating behavior, as appropriate. Participants in the national samples then installed Laddr on their own smartphone, and participants in the locally recruited passive-sensing samples had Laddr installed on a study-provided smartphone. All participants were asked to use Laddr at least daily.

During the 14-day study period, participants were queried about momentary self-regulation, emotional state, and social and environmental context (both samples; 8 general context questions); recent smoking and exposure to smoking cues (smoking sample only; 14 different smoking-specific contexts); and recent eating, binge eating, and exposure to binge-eating cues (binge-eating sample only; 12 different binge-eating-specific contexts). In addition to delivering the momentary intervention, Laddr collected the momentary assessments. Participants were prompted four times per day; prompts were delivered during random times within four windows based on their stated waking hours (e.g., 8–11:30 AM, 11:30 AM−3 PM, 3–6:30 PM, 6:30–10 PM) and delivered at least 1 h apart.

Participants were compensated for completing baseline and momentary assessments, and for interacting with the Laddr application, and could receive up to $175 over the 2-week study period. Compensation was determined according to the following schedule: $20 per participant for all baseline surveys; $1 per momentary assessment completed (4 momentary assessments per day for 14 days, maximum compensation of $56); $2 per day for Laddr activities (at least 5 minutes of engagement per day for 14 days, maximum compensation of $28); $20 bonus per week for completing at least 25 of the 28 momentary assessments that week (2 weeks, maximum compensation of $40); and $31 for data plan reimbursement.

Participants also participating in the passive-sensing mobile data collection followed a slightly adapted compensation schedule for activities related to mobile data collection for a maximum total compensation of $250.

### Baseline Measures

The baseline questionnaire measured demographic information (e.g., age, gender, ethnicity, race, education, income), height and weight for BMI, smoking-related questions (e.g., number of quit attempts), and binge-eating-related questions (e.g., currently on a diet to lose weight). The eight-item Patient Health Questionnaire depression scale (PHQ-8; (36) and Reward-based Eating Drive scale (RED-13; (37) were also collected in both samples.

In the smoking sample, participants completed the Fagerström Test for Nicotine Dependence (38). In the binge-eating sample, binge-eating disorder (BED) was screened *via* the binge-eating questions from the QEWP-5 (27).

In addition, several self-regulation questionnaires were administered. The questionnaires used in this study were a subset of questionnaires selected in the overarching self-regulation project as measures that putatively measure aspects of self-regulation (1). The subset included any scale from which momentary self-regulation subscale items were derived (Scherer et al., under review). These were collected to describe the sample with respect to self-regulation and to confirm the associations between aspects of self-regulation at the trait level and those at the momentary level. The baseline self-regulation questionnaires were: (1) Short Self-Regulation Questionnaire [SSRQ; (39)]; (2) both subscales of the Emotion Regulation Questionnaire (ERQ)—cognitive reappraisal and expressive suppression (40); (3) all five subscales of the Five Facet Mindfulness Questionnaire (FFMQ)—observing, describing, acting with awareness, non-judging, and non-reactivity (41); (4) Mindful Attention Awareness Scale [MAAS; (42)]; (5) all four subscales of the Selection-Optimization-Compensation Questionnaire, short version (SOC)—elective selection, loss-based selection, optimization, and compensation (43); (6) two of the five subscales of the UPPS-P Impulsive Behavior Scale—lack of premeditation and lack of perseverance (44–46); and (7) one of the three subscales of the Eysenck I-7 Impulsiveness and Venturesomeness Questionnaire—venturesomeness (47).

### Momentary Measures

#### Momentary Self-Regulation Scale

At each EMA prompt, participants were queried about momentary self-regulation using a novel momentary self-regulation scale. This metric operationalizes and empirically examines self-regulatory processes and dynamics in a naturalistic setting and can be implemented through mobile devices for EMA. This measure allows for testing hypotheses related to momentary self-regulation (Scherer et al., in preparation).

Developing this momentary self-regulation scale was an initial step in the overarching SOBC project. In developing this metric, we identified underlying constructs of self-regulation *via* factor analyses on 23 existing scales (594 items) measuring self-regulation. Focusing on items from various scales that loaded highly on each underlying construct, we modified the language of the items to focus on a momentary state rather than an individual trait. For example, if the existing scale item was “I've kept my emotions to myself,” the wording was modified to “Since the last prompt, I've kept my emotions to myself.” From a set of momentary items piloted on 53 participants, and through further factor analysis, a 12-item momentary self-regulation scale was developed. The scale demonstrated construct validity with a high level of association between the trait-level and moment-level items. The scale also demonstrated both intra- and interindividual variability, demonstrating that individuals differed on their mean level of self-regulation and within individual, there was significant variability in levels of self-regulation. The momentary self-regulation scale consists of four subscales, each with three items: (1) momentary perseverance, (2) momentary sensation seeking, (3) momentary self-judgment, and (4) momentary mindfulness. This newly developed scale allows for the study of self-regulation on a momentary level and was a key assessment tool used in the present study.

Briefly, momentary perseverance is comprised of items on setting goals and tracking progress toward them, and continuing working on projects until they are finished or a goal has been achieved. Momentary sensation seeking is comprised of items relating to taking and enjoying taking risks. If creating a total momentary self-regulation score, this subscale is reverse coded as it negatively associates with the construct of self-regulation. Momentary self-judgement items measure negative judgments about self or one's emotions. Like sensation seeking, this subscale is reverse coded if creating a momentary self-regulation total score. Finally, momentary mindfulness items measure being attentive and mindful to task.

The 12 items of the momentary self-regulation scale were assessed on each momentary assessment. As described above, the scale was developed to measure the underlying constructs of existing trait-level self-regulation scales as well as to detect momentary fluctuations in these constructs. A full description of the scale development and pilot study can be found in Scherer et al. under review.

#### Momentary Context Measures

At each prompt, participants were asked whether they were alone or with others and where they were (home, friend's or family member's house, car, work or school, cafeteria/restaurant/bar, outside, other). Context items were based on earlier work on assessing participant context using EMA (48–51). Response options were grouped due to small counts to facilitate modeling. In addition, momentary positive and negative affect, stress, and tiredness were assessed *via* several 7-point Likert items, loosely based on items developed by Wilhelm and Schoebi (52). These general context measures were assessed to evaluate how various daily contexts (without a direct relation to a health risk behavior) may influence momentary self-regulation.

In addition to the general context measures, sample-specific context measures were asked of each sample. These context measures were assessed to evaluate how contexts that are more directly related to a health behavior may influence momentary self-regulation. Participants in the smoking sample were asked a series of questions about their smoking behavior as well as recent eating and recent alcohol and drug use. They were also asked about several smoking cues, including seeing others smoke, seeing cigarettes, and smelling smoke. Participants who smoked were asked about current access to cigarettes, likelihood of smoking in the next 4 h, temptation to smoke, urge to smoke, and restraint in smoking. These items were included to assess the associations with momentary self-regulation; the items were informed by earlier EMA smoking studies (48, 49, 53) and an EMA eating study (54).

Participants in the binge-eating sample were asked a series of questions about their eating behavior, including binge eating. They were also asked about seeing or smelling food, ease of access to food, likelihood of binge eating in the next 4 h, urge to binge eat, hunger, and restraint in eating. As with the smoking sample, the goal of including these items was to assess their association with momentary self-regulation. Many of the binge-eating items were broadly derived from several studies of disordered eating, body weight, and affect (54–59).

Finally, based on Laddr usage data, we calculated whether a participant had used Laddr within the past hour and within the past 12 h of responding to the prompt. This measure was calculated to assess whether Laddr use was associated with momentary self-regulation, and whether Laddr use modified the relationships between contexts and momentary self-regulation.

#### Daily Measures

Morning questionnaires for the smoking sample included questions on desire to smoke that day and motivation to avoid smoking that day. Morning questionnaires for the binge-eating sample included questions on desire to binge eat that day and motivation to avoid binge eating that day. Participants in the binge-eating sample were presented items from the Three-Factor Eating Questonnaire-R18 (60) in the evening to assess binge-eating behavior during the day. Both morning and evening daily measures were included to assess whether they were associated with the level of momentary self-regulation during that day.

### Statistical Analyses

Participant characteristics were evaluated *via* descriptive statistics. The smoking and binge-eating samples were compared *via* chi-squared tests for categorical variables and *t*-tests for continuous variables.

All participants who completed at least one momentary questionnaire are included in the analyses of momentary measures. Descriptive statistics were computed for responses to momentary context questions.

The associations between baseline self-regulation measures and the momentary self-regulation subscales were examined to confirm their association (results included in Supplementary Tables 4–6). The primary results for this manuscript are the evaluation of associations between momentary context questions and the momentary self-regulation subscales. These were evaluated *via* mixed-effects models. Each model included a fixed effect for the baseline measure or momentary context question as well as a time variable to account for changes in momentary self-regulation over the course of the study. Each model also included an individual-level intercept term to account for the non-independence of repeated observations from the same individual. Regression parameter estimates are provided for the momentary context question along with standard error and *p*-value. In addition to the primary evaluation, models with an interaction between recent Laddr use (defined as use within 12 h of momentary responses) and momentary contexts were fit to examine how Laddr use modified the relationship between momentary contexts and momentary self-regulation. In the results for the interaction models, a type III *p*-value from the context-by-Laddr term is presented to test for effect modification. Linear combinations of mixed model parameter estimates from the interaction model are used to estimate the association between context and momentary self-regulation within moments with recent Laddr use (within the past 12 h) and within moments without recent Laddr use (within the past 12 h). A significant *p*-value (<0.05) indicates a statistically significant association between the momentary context measure and the momentary self-regulation subscale.

The association between general context measures and momentary self regulation is presented in a combined sample of the smoking and binge eating sample, while the association between condition-specific context measures and momentary self-regulation is examined separately for each sample. The association between the general context measures and momentary self-regulation was also assessed in the smoking sample and the binge-eating sample and results are in (Supplementary Tables 7–10).

Due to the exploratory nature of the study and the study's primary focus on assessing if self-regulation can be measured and engaged in the moment in two exemplar populations with health risk behavior, many statistical comparisons were made, but no adjustment was made for multiple comparisons. Even if no true associations exist, we would expect about 5% of the statistical tests to be significant, so individual significant results should be interpreted with caution and replicated in further studies.

The study protocol and statistical analysis plan were preregistered on ClinicalTrials.gov (Identifier: NCT03352713). All analyses were performed using SAS® software version 9.4 (61).

## Results

### Study Participants

**Figure 1:**
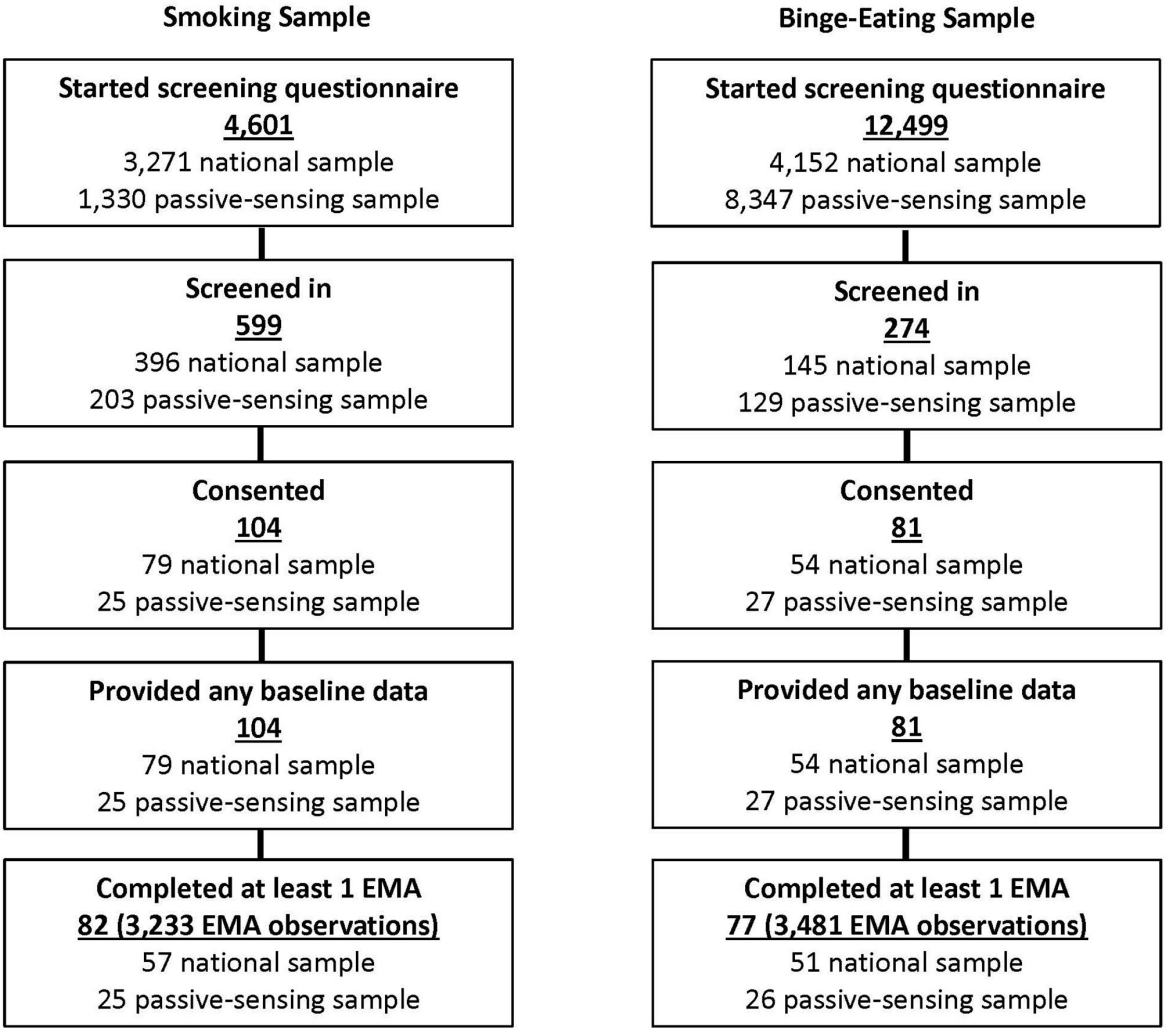
Participant flow diagram.

A total of 4,601 participants were screened for the smoking sample, and 12,499 were screened for the binge-eating sample. Of those, 599 and 274 screened in, respectively, and 104 and 81 participants consented, respectively. The primary reasons for screening out of the smoking sample were BMI <17 or ≥27 kg/m^2^, age <21 or >50 years, and not completing the screening questionnaire. For the binge-eating sample, the primary reasons for screening out were not meeting the BED criteria, BMI <27 or >45 kg/m^2^, and not completing the screening questionnaire. All participants provided baseline data, and 82 participants from the smoking sample and 77 participants from the binge-eating sample provided at least one response to a momentary questionnaire. Participants in the smoking sample contributed a total of 3,233 momentary questionnaires, and participants in the binge-eating sample contributed a total of 3,481 momentary questionnaires. See Figure 1.

### Participant Characteristics, Baseline Measures, and EMA Response Rates

The mean age of the combined sample was 35.2 years (7.9 SD), and the majority of participants were female (78.4%). A large majority of participants were white (89.2%), which is, in part, due to the racial distribution in the location of the local sample recruitment. A large percentage of participants had some college or an associate degree (43.2%). An additional 22.7% completed a bachelor's degree, and 15.7% had an advanced (graduate) degree. The smoking sample and binge-eating sample differed in age, gender, BMI, education, and income, with the binge-eating sample having higher BMI, education level, and income. The smoking sample was slightly older (36.6 years vs. 33.5 years) and consisted of a larger proportion of males. See Table 1.

**Table 1:** Participant demographics in the combined, smoking, and binge-eating samples.

	**Combined sample**			**Smoking sample**			**Binge-eating sample**			***p*-value**
	***N*** **=** **185**			***N*** **=** **104**			***N*** **=** **81**			
	***N***	**Mean** **%**	**SD**	***N***	**Mean** **%**	**SD**	***N***	**Mean** **%**	**SD**	
Age	185	35.2	7.9	104	36.6	7.7	81	33.5	7.9	0.008
Gender										0.008
Male	37	20.0%		29	27.9%		8	9.9%		
Female	145	78.4%		74	71.2%		71	87.7%		
Non-binary/third gender	3	1.6%		1	0.9%		2	2.5%		
Race										0.15
Black/African American	12	6.5%		4	3.8%		8	9.9%		
White	165	89.2%		94	90.4%		71	87.7%		
American Indian/Alaska Native/Asian/Native Hawaiian/Pacific Islander/other/more than one race	8	4.3%		6	5.8%		2	2.5%		
Hispanic ethnicity	6	3.2%		2	1.9%		4	4.9%		0.41
Body mass index	185	28.3	6.8	104	23.3	2.5	81	34.8	4.7	<0.001
Diabetes	2	1.1%		1	1.0%		1	1.2%		1.0
Education										<0.001
Some high school or less	5	2.7%		5	4.8%		0	0.0%		
High school diploma or General Educational Development (GED)	29	15.7%		27	26.0%		2	2.5%		
Some college/associate's (2-year) degree	80	43.2%		53	51.0%		27	33.3%		
Bachelor's (4-year) degree	42	22.7%		14	13.5%		28	34.6%		
Advanced (graduate) degree	29	15.7%		5	4.8%		24	29.6%		
Income										<0.001
$0-$25,999	34	18.8%		23	23.0%		11	13.6%		
$26,000-$51,999	66	36.5%		47	47.0%		19	23.5%		
$52,000-$74,999	40	22.1%		19	19.0%		21	25.9%		
$75,000+	41	22.7%		11	11.0%		30	37.0%		
Marital status										0.15
Single (never married)	65	35.1%		33	31.7%		32	39.5%		
Married	88	47.6%		47	45.2%		41	50.6%		
Separated	7	3.8%		6	5.8%		1	1.2%		
Widowed	2	1.1%		2	1.9%		0	0.0%		
Divorced	23	12.4%		16	15.4%		7	8.6%		

The binge-eating sample had higher mean PHQ-8 and RED-13 scores than the smoking sample. In the smoking sample, over half (54.8%) of the participants indicated they were currently trying to cut down or quit smoking, and about 80% smoked more than 10 cigarettes per day. Only 25.9% of the binge-eating sample was currently on a diet or trying to lose weight. Baseline self-regulation measures also differed between the groups indicating some differences in trait-level self regulation between the two samples. Scores on the SSRQ, ERQ cognitive appraisal, FFMQ acting with awareness, FFMQ non-judging, FFMQ non-reactivity, SOC optimization, and SOC compensation were all higher in the smoking sample. Scores on the MAAS and UPPS-P lack of perseverance (where a higher score in the latter represents lower self-regulation) were higher in the binge-eating sample. See Table 2.

**Table 2:** Baseline, study-specific measures in the combined, smoking, and binge-eating samples.

	**Combined sample**			**Smoking sample**			**Binge-eating sample**			***p*-value**
	***N*** **=** **185**			***N*** **=** **104**			***N*** **=** **81**			
	***N***	**Mean** **%**	**SD**	***N***	**Mean** **%**	**SD**	***N***	**Mean** **%**	**SD**	
Reward-based Eating Drive scale (RED-13)	184	25.0	13.9	104	15.0	9.2	80	37.9	5.7	<0.001
PHQ-8	185	7.1	5.3	104	5.3	5.0	81	9.3	4.6	<0.001
**Smoking behavior**										
Currently trying to quit/cut down on smoking				57	54.8%					
Number of cigarettes per day				104	18.3	7.2				
≤ 10				21	20.2%					
11–20				60	57.7%					
>20				23	22.1%					
Number of quit attempts—lifetime				25	6.1	10.2				
0				2	8.0%					
1–10				21	84.0%					
>10				2	8.0%					
Quit attempt in the past 12 months				11	44.0%					
Fagerström				102	5.3	2.0				
**Binge-eating behavior**										
Currently on a diet/trying to lose weight							21	25.9%		
Greatest weight							81	235.8	50.5	
**Self-regulation measures**										
SSRQ	182	102.5	17.1	102	107.7	17.6	80	95.9	14	<0.001
ERQ cognitive reappraisal	182	28.2	7.2	102	29.9	6.8	80	26.1	7.2	<0.001
ERQ expressive suppression	182	15.1	5.6	102	15.0	5.5	80	15.2	5.7	0.78
FFMQ acting with awareness	182	24.4	6.2	102	26.3	6.0	80	22.0	5.7	<0.001
FFMQ observing	182	26.8	5.3	102	27.1	5.0	80	26.4	5.6	0.37
FFMQ describing	182	27.0	6.8	102	27.0	6.8	80	27.0	6.9	0.90
FFMQ non-judging	182	23.6	7.2	102	25.5	6.9	80	21.2	6.9	<0.001
FFMQ non-reactivity	182	20.8	4.6	102	22.1	4.0	80	19.1	4.7	<0.001
MAAS	182	3.4	0.9	102	3.1	0.9	80	3.9	0.8	<0.001
SOC elective selection	181	0.7	0.9	101	0.7	0.9	80	0.7	0.9	0.71
SOC loss-based selection	181	1.3	1.0	101	1.3	0.9	80	1.3	1.1	0.98
SOC optimization	181	1.5	1.2	101	1.9	1.1	80	1.1	1.2	<0.001
SOC compensation	181	1.4	1.0	101	1.6	1.0	80	1.2	1.0	0.004
UPPS-P lack of premeditation	181	2.0	0.6	101	2.0	0.5	80	1.9	0.6	0.40
UPPS-P lack of perseverance	181	2.1	0.6	101	2.0	0.6	80	2.3	0.6	0.002
Eysenck I-7 venturesomeness	181	7.8	4.4	101	8.4	4.5	80	7.1	4.3	0.06
**EMA completion**										
Contributed EMA data	159	85.9		82	78.8		77	95.1		0.002
Number of days contributed EMA data	159	13.4	3.5	82	13.1	4.4	77	13.7	2.1	0.34
Average number of EMA reports per day	159	3.1	0.8	82	2.9	0.9	77	3.3	0.7	0.01

*Gray shading indicates cells not relevant for the particular sample*.

A higher proportion of participants in the binge-eating sample contributed momentary data (95.1% vs. 78.8%), and those in the binge-eating sample contributed slightly more momentary reports per day than those in the smoking sample (3.3 vs. 2.9). Among those who contributed momentary data, the number of days contributed were similar between the two samples. See Table 2.

### EMA Measures

Descriptive statistics for momentary measures are presented in Supplementary Tables 1–3.

### Associations Between General Momentary Context Measures and Momentary Self-Regulation Subscales in the Combined Sample

In the combined sample, the associations between momentary context measures asked of both samples were evaluated for their relationship to momentary self-regulation measures. See Table 3. Momentary **perseverance** was significantly higher when in the car or at work (vs. home), and was positively associated with momentary positive affect, and negatively associated with being alone, momentary negative affect, momentary stress level, and momentary tiredness. Momentary **sensation seeking** was positively associated with momentary positive affect and was, on average, higher when in “other” locations (vs. home), and lower when alone. It was negatively associated with momentary negative affect, and momentary stress level, with higher negative affect and higher stress associated with lower momentary sensation seeking. Momentary **self-judgment** was positively associated with momentary negative affect, momentary stress level, and momentary tiredness, and was, on average, lower when in “other” locations or at work (vs. home). It was also negatively associated with momentary positive affect. Momentary **mindfulness** was positively associated with momentary positive affect and was, on average, higher when in “other” locations (vs. home), and lower when at work (vs. home), and negatively associated with momentary negative affect, momentary stress level, and momentary tiredness. Generally, it appears that being alone or in specific locations may be associated with momentary self-regulation. But different locations may influence different aspects (subscales) of momentary self-regulation. In contrast, positive and negative affect, stress and tireness were associated more uniformly with all aspects of momentary self-regulation.

**Table 3:** Momentary context measures, association with momentary self-regulation in the combined sample.

	**Momentary perseverance**				**Momentary sensation seeking**				**Momentary self-judgment**				**Momentary mindfulness**			
**Momentary context**	**β**	**SE**	**DF**	***p*-value**	**β**	**SE**	**DF**	***p*-value**	**β**	**SE**	**DF**	***p*-value**	**β**	**SE**	**DF**	***p*-value**
Alone	**−0.06**	**0.02**	**6,553**	**0.01**	**−0.06**	**0.01**	**6,552**	**<0.0001**	0.01	0.02	6,553	0.62	**–**0.02	0.02	6,552	0.35
At this moment, location:																
Car	**0.22**	**0.04**	**6,551**	**<0.0001**	0.01	0.02	6,550	0.66	**–**0.01	0.03	6,551	0.67	**–**0.06	0.03	6,550	0.08
Other	0.05	0.04	6,551	0.16	**0.11**	**0.02**	**6,550**	**<0.0001**	**–**0.08	0.03	6,551	0.01	**0.08**	**0.03**	**6,550**	**0.01**
Work	**0.27**	**0.03**	**6,551**	**<0.0001**	**–**0.002	0.02	6,550	0.92	**−0.08**	**0.02**	**6,551**	**<0.001**	**−0.16**	**0.02**	**6,550**	**<0.0001**
Home (reference)																
Right now: positive affect	**0.21**	**0.01**	**6,553**	**<0.0001**	**0.05**	**0.01**	**6,552**	**<0.0001**	**−0.13**	**0.01**	**6,553**	**<0.0001**	**0.14**	**0.01**	**6,552**	**<0.0001**
Right now: negative affect	**−0.14**	**0.01**	**6,553**	**<0.0001**	**−0.03**	**0.01**	**6,552**	**<0.0001**	**0.19**	**0.01**	**6,553**	**<0.0001**	**−0.15**	**0.01**	**6,552**	**<0.0001**
Right now: how stressed?	**−0.05**	**0.01**	**6,553**	**<0.0001**	**−0.02**	**0.005**	**6,552**	**<0.0001**	**0.09**	**0.01**	**6,553**	**<0.0001**	**−0.08**	**0.01**	**6,552**	**<0.0001**
Right now: how tired?	**−0.07**	**0.01**	**6,553**	**<0.0001**	**–**0.004	0.004	6,552	0.37	**0.04**	**0.01**	**6,553**	**<0.0001**	**−0.06**	**0.01**	**6,552**	**<0.0001**
Used laddr (≤12 h)	0.01	0.02	6,553	0.65	**–**0.004	0.01	6,552	0.80	0.002	0.02	6,553	0.92	**–**0.03	0.02	6,552	0.10
Used laddr (≤1 h)	**–**0.02	0.03	6,553	0.50	0.01	0.02	6,552	0.62	0.05	0.03	6,553	0.08	**–**0.05	0.03	6552	0.11

*Parameter estimates from mixed-effects models. Bold values indicate p-values ≤ 0.05*.

While recent Laddr use (within 12 h and within 1 h) was not associated with the momentary self-regulation subscales (Table 3), when examining the model with an interaction between Laddr use and context, we see that recent Laddr use (within the past 12 h) significantly modified the association between momentary **mindfulness** and location (Table 4). Momentary mindfulness was lower at work than at home, and there was a larger difference in momentary mindfulness between work and home when Laddr had been used in the past 12 h. One possible reason for this may be that using Laddr had a stronger positive influence on momentary mindfulness at home than it did when someone was working. Or, momentary mindfulness while at work is generally lower (as seen in the main results), and less malleable with interventions than it would be at home.

**Table 4:** Momentary context measures, association with momentary self-regulation by recent Laddr use (≤12 h) in the combined sample.

**Association between momentary context and momentary self-regulation measures** **Presented separately by recent Laddr use (≤12 h)**	**Momentary perseverance**				**Momentary sensation seeking**				**Momentary self-judgment**				**Momentary mindfulness**			
	β^*^	**SE**	**DF**	***p*-value**	β^*^	**SE**	**DF**	***p*-value**	β^*^	**SE**	**DF**	***p*-value**	β^*^	**SE**	**DF**	***p*-value**
**Momentary context**																
Alone				1.00				0.89				0.72				0.83
Laddr use (≤12 h)	−0.05	0.03	6,552	0.13	−0.06	0.02	6,551	0.002	0.003	0.03	6,552	0.92	−0.01	0.03	6,551	0.63
No Laddr (≤12 h)	−0.05	0.03	6,552	0.11	−0.06	0.02	6,551	<0.001	0.02	0.02	6,552	0.54	−0.02	0.03	6,551	0.42
At this moment, location:																
Car				0.90				0.33				0.72				0.29
Laddr use (≤12 h)	0.21	0.06	6,548	<0.001	−0.01	0.03	6,547	0.68	−0.02	0.04	6,548	0.58	−0.09	0.05	6,547	0.05
No Laddr (≤12 h)	0.22	0.05	6,548	<0.0001	0.03	0.03	6,547	0.32	−0.003	0.04	6,548	0.94	−0.02	0.04	6,547	0.58
Other				0.51				0.18				0.67				0.21
Laddr use (≤12 h)	0.07	0.05	6,548	0.13	0.14	0.03	6,547	<0.0001	−0.07	0.04	6,548	0.09	0.04	0.04	6,547	0.30
No Laddr (≤12 h)	0.03	0.05	6,548	0.55	0.08	0.03	6,547	0.005	−0.09	0.04	6,548	0.02	0.11	0.04	6,547	0.01
Work				0.06				0.06				0.87				**0.04**
Laddr use (≤12 h)	0.22	0.04	6,548	<0.0001	−0.03	0.02	6,547	0.17	−0.08	0.03	6,548	0.01	–**0.21**	**0.03**	**6,547**	**<0.0001**
No Laddr (≤12 h)	0.32	0.04	6,548	<0.0001	0.03	0.02	6,547	0.25	−0.08	0.03	6,548	0.01	–**0.12**	**0.03**	**6,547**	**<0.001**
Home (reference)																
Right now: positive affect				0.65				0.09				0.08				0.18
Laddr use (≤12 h)	0.19	0.01	6,552	<0.0001	0.06	0.01	6,551	<0.0001	−0.15	0.01	6,552	<0.0001	0.14	0.01	6,551	<0.0001
No Laddr (≤12 h)	0.20	0.01	6,552	<0.0001	0.05	0.01	6,551	<0.0001	−0.12	0.01	6,552	<0.0001	0.13	0.01	6,551	<0.0001
Right now: negative affect				0.38				0.44				0.93				0.11
Laddr use (≤12 h)	−0.15	0.01	6,552	<0.0001	−0.03	0.01	6,551	<0.001	0.19	0.01	6,552	<0.0001	−0.16	0.01	6,551	<0.0001
No Laddr (≤12 h)	−0.14	0.01	6,552	<0.0001	−0.02	0.01	6,551	0.01	0.19	0.01	6,552	<0.0001	−0.14	0.01	6,551	<0.0001
Right now: how stressed?				0.82				0.23				0.26				0.06
Laddr use (≤12 h)	−0.05	0.01	6,552	<0.0001	−0.03	0.01	6,551	<0.0001	0.09	0.01	6,552	<0.0001	−0.10	0.01	6,551	<0.0001
No Laddr (≤12 h)	−0.05	0.01	6,552	<0.0001	−0.02	0.01	6,551	0.001	0.08	0.01	6,552	<0.0001	−0.08	0.01	6,551	<0.0001
Right now: how tired?				0.68				0.07				0.53				0.11
Laddr use (≤12 h)	−0.07	0.01	6,552	<0.0001	−0.01	0.01	6,551	0.07	0.04	0.01	6,552	<0.0001	−0.07	0.01	6,551	<0.0001
No Laddr (≤12 h)	−0.06	0.01	6,552	<0.0001	0.003	0.01	6,551	0.63	0.04	0.01	6,552	<0.0001	−0.06	0.01	6,551	<0.0001

*Parameter estimates from mixed-effects models with a context-by-Laddr interaction term*.

* *Regression coefficients presented in each row represent the association between the context measure and the momentary self-regulation outcome. For each context, they are presented for moments when Laddr had been used recently, and when Laddr had not been used recently. Bold values indicate p-values ≤ 0.05*.

Results in the smoking (Supplementary Tables 7, 8) and binge eating samples (Supplementary Tables 9, 10) did not differ meaningfully from the combine sample results.

### Associations Between Momentary Context Measures and Momentary Self-Regulation Subscales in the Smoking Sample

#### Smoking-Specific Context Measures

In the smoking sample, smoking-related contexts were evaluated for their association with momentary self-regulation (Table 5). Momentary difficulty in accessing cigarettes, momentary caffeine intake, and momentary restraint in smoking were all positively associated with momentary **perseverance**, indicating these external and internal contexts engaged the perseverance aspect of an individual's self-regulation. Momentary temptation to smoke and momentary urge to smoke, on the other hand, were both negatively associated with momentary **perseverance**, indicating these challenges, indeed, negatively impacted an individual's perseverance in the moment. Having seen someone smoke, smelling smoke, and drinking alcohol were all positively associated with momentary **sensation seeking**, supporting these contexts as cues that engage an individual's desire for increased sensation seeking. Having seen someone smoke, smelling smoke, eating, drinking alcohol, momentary temptation to smoke, and momentary urge to smoke were all positively associated with momentary **self-judgment**, showing how these smoking cues influence an individual's negative self-view. Whereas a greater feeling of momentary restraint in smoking was associated with lower levels of momentary **self-judgment**, indicating a more positive self view with higher levels of restraint. Interestingly, a higher daily desire to smoke (which was measured in the morning) was associated with lower momentary **mindfulness** (measured throughout the day), indicating the desire to smoke may influence mindfulness. When an individual indicated a higher likelihood of smoking within the next 4 h and and higher momentary restraint in smoking, this was associated with increased momentary **mindfulness**. Momentary difficulty in accessing cigarettes, momentary temptation to smoke, and momentary urge to smoke were all associated with lower levels of momentary **mindfulness**. While still exploratory, taken together, these results indicate that smoking-related context is associated with momentary self-regulation levels. It appears temptation, urge, and restraint are all associated with several aspects of momentary self-regulation: perseverance, self-judgement, and mindfulness. Interestingly, smoking cues (seeing or smelling smoke, and drinking alcohol), appear to be the only contexts that may influence momentary sensation seeking, but these may also influence an individual's self-judgement. Also of note, was that smoking since the last prompt was not associated with any of the momentary self-regulation subscales.

**Table 5:** Momentary context measures, association with momentary self-regulation in the smoking sample.

	**Momentary perseverance**				**Momentary sensation seeking**				**Momentary self-judgment**				**Momentary mindfulness**			
	**β**	**SE**	**DF**	***p*-value**	**β**	**SE**	**DF**	***p*-value**	**β**	**SE**	**DF**	***p-*value**	**β**	**SE**	**DF**	***p*-value**
**Morning-only questions:**																
Desire to smoke today	0.02	0.01	2,742	0.14	−0.004	0.01	2,742	0.51	0.002	0.01	2,742	0.77	–**0.02**	**0.01**	**2,741**	**0.004**
Motivated to avoid smoking	0.01	0.01	2,742	0.43	−0.003	0.01	2,742	0.57	0.005	0.01	2,742	0.42	−0.01	0.01	2,741	0.17
**Questions on all prompts:**																
Seen other smoke	0.05	0.04	3,149	0.13	**0.05**	**0.02**	**3,149**	**0.02**	0.05	0.03	3,149	0.04	−0.04	0.03	3,148	0.13
Seen cigarette	0.03	0.05	3,149	0.57	0.01	0.03	3,149	0.79	0.004	0.04	3,149	0.91	−0.06	0.04	3,148	0.14
Smelled smoke	−0.03	0.04	2,935	0.50	**0.07**	**0.02**	**2,935**	**0.002**	**0.06**	**0.03**	**2,935**	**0.03**	-0.03	0.03	2,934	0.36
Hard to access cigarettes	**0.06**	**0.02**	**3,149**	**<0.001**	−0.01	0.01	3,149	0.45	0.01	0.01	3,149	0.28	–**0.03**	**0.01**	**3,148**	**0.03**
Eaten	0.03	0.03	3,149	0.41	0.01	0.02	3,149	0.63	**0.05**	**0.02**	**3,149**	**0.03**	−0.04	0.02	3,148	0.14
Alcohol	0.07	0.07	3,149	0.33	**0.09**	**0.04**	**3,149**	**0.02**	**0.19**	**0.05**	**3,149**	**<0.0001**	0.02	0.05	3,148	0.75
Caffeine	**0.08**	**0.03**	**3,149**	**0.03**	−0.02	0.02	3,149	0.30	−0.02	0.02	3,149	0.38	−0.02	0.03	3,148	0.54
Likely smoke in next 4 h	0.03	0.02	3,149	0.11	0.01	0.01	3,149	0.16	−0.01	0.01	3,149	0.30	**0.04**	**0.01**	**3,148**	**0.001**
Temptation to smoke	**−0.03**	**0.01**	**3,149**	**0.01**	0.01	0.01	3,149	0.07	**0.03**	**0.01**	**3,149**	**<0.001**	**−0.02**	**0.01**	**3,148**	**0.02**
Urge to smoke	**−0.03**	**0.01**	**3,149**	**0.01**	0.01	0.01	3,149	0.053	**0.03**	**0.01**	**3,149**	**0.002**	**−0.03**	**0.01**	**3,148**	**0.01**
Restraint in smoking	**0.03**	**0.01**	**3,149**	**<0.0001**	0.004	0.004	3,149	0.39	**−0.01**	**0.01**	**3,149**	**0.01**	**0.01**	**0.01**	**3,148**	**0.01**
Smoked since last prompt	0.09	0.06	3,149	0.12	0.02	0.03	3,149	0.50	0.002	0.04	3,149	0.96	0.08	0.05	3,148	0.10

*Parameter estimates from mixed-effects models. Bold values indicate p-values ≤ 0.05*.

Recent Laddr use modified several of the relationships between smoking-specific context measures and momentary self-regulation, as seen in the model including an interaction term between Laddr use (within the past 12 h) and context (Table 6). Following recent Laddr use, the association between difficulty accessing cigarettes and increased momentary **perseverance** was smaller in magnitude than when Laddr had not been recently used. This is consistent with a weakened link between context and momentary perseverance with Laddr use. With recent Laddr use, seeing others smoke was significantly associated with higher momentary **sensation seeking**, but when Laddr had not been recently used, there was no significant association. This pattern could be interpreted as Laddr increasing an individual's awareness of momentary smoking cues. For recent alcohol use, momentary temptation to smoke, and momentary urge to smoke, a significant positive association existed with momentary **self-judgment** when following recent Laddr use, but the association was not significant without recent Laddr use. Again, this may be that indicate that Laddr usage affected how an individual is influenced by smoking cues. A similar pattern was seen in the association between temptation and urge to smoke and momentary **mindfulness**—with the association significant only following recent Laddr use. Finally, the direction of the association between alcohol use and momentary **mindfulness** differed for prompts with (negative) vs. without (positive) recent Laddr use. These analyses are exploratory, thus over-interpretting a particular significant associations is inappropriate. However, the collective finding that in multiple instances, the association between a smoking-specific context and momentary self-regulation differed when a health behavior intervention was vs. was not used, may offer preliminary indication that interventions to promote health behavior have the potential to interrupt context-specific influences on self-regulation or modify their effect on an individual's self-regulation.

**Table 6:** Momentary context measures, association with momentary self-regulation by recent Laddr use (≤12 h) in the smoking sample.

**Association between momentary context and momentary self-regulation measures** **Presented separately by recent Laddr use (≤12 h)**	**Momentary perseverance**				**Momentary sensation seeking**				**Momentary self-judgment**				**Momentary mindfulness**			
	β^*^	**SE**	**DF**	***p*-value**	β^*^	**SE**	**DF**	***p*-value**	β^*^	**SE**	**DF**	***p*-value**	β^*^	**SE**	**DF**	***p*-value**
**Morning-only questions:**																
Desire to smoke today				0.78				0.19				0.15				0.06
Laddr use (≤12 h)	−0.004	0.01	2,741	0.79	−0.01	0.01	2,741	0.49	0.01	0.01	2,741	0.15	−0.04	0.01	2,740	<0.001
No Laddr (≤12 h)	0.0009	0.01	2,741	0.94	0.01	0.01	2,741	0.35	−0.003	0.01	2,741	0.74	−0.01	0.01	2,740	0.22
Motivated to avoid smoking				0.45				0.51				0.17				0.25
Laddr use (≤12 h)	0.03	0.01	2,741	0.02	−0.01	0.01	2,741	0.13	−0.003	0.01	2,741	0.62	−0.02	0.01	2,740	0.08
No Laddr (≤12 h)	0.02	0.01	2,741	0.13	−0.005	0.01	2,741	0.42	0.01	0.01	2,741	0.26	−0.004	0.01	2,740	0.63
**Questions on all prompts:**																
Seen other smoke				0.74				**0.003**				0.10				0.10
Laddr use (≤12 h)	0.03	0.05	3,148	0.61	**0.11**	**0.03**	**3,148**	**<0.001**	0.10	0.04	3,148	0.01	−0.09	0.04	3,147	0.03
No Laddr (≤12 h)	0.05	0.05	3,148	0.31	0.003	0.03	3,148	0.90	0.02	0.03	3,148	0.49	−0.01	0.04	3,147	0.85
Seen cigarette				0.39				0.17				0.66				0.24
Laddr use (≤12 h)	−0.07	0.07	3,148	0.30	0.06	0.04	3,148	0.12	0.003	0.05	3,148	0.95	−0.11	0.05	3,147	0.05
No Laddr (≤12 h)	0.01	0.07	3,148	0.94	−0.01	0.04	3,148	0.81	0.03	0.05	3,148	0.52	−0.03	0.05	3,147	0.63
Smelled smoke				0.49				**0.05**				**0.05**				0.10
Laddr use (≤12 h)	−0.10	0.05	2,934	0.08	**0.12**	**0.03**	**2,934**	**<0.0001**	**0.11**	**0.04**	**2,934**	**0.002**	−0.08	0.04	2,933	0.07
No Laddr (≤12 h)	−0.05	0.05	2,934	0.35	0.05	0.03	2,934	0.09	0.03	0.04	2,934	0.46	0.01	0.04	2,933	0.83
Hard to access cigarettes				**0.01**				0.44				0.56				0.21
Laddr use (≤12 h)	**0.05**	**0.02**	**3,148**	**0.04**	−0.01	0.01	3,148	0.61	0.004	0.02	3,148	0.78	−0.02	0.02	3,147	0.35
No Laddr (≤12 h)	**0.13**	**0.03**	**3,148**	**<0.0001**	−0.02	0.02	3,148	0.17	0.02	0.02	3,148	0.34	−0.05	0.02	3,147	0.02
Eaten				0.49				0.91				0.90				0.23
Laddr use (≤12 h)	0.01	0.05	3,148	0.86	0.01	0.03	3,148	0.72	0.05	0.03	3,148	0.14	−0.003	0.04	3,147	0.93
No Laddr (≤12 h)	0.05	0.04	3,148	0.22	0.01	0.02	3,148	0.81	0.04	0.03	3,148	0.14	−0.06	0.03	3,147	0.06
Alcohol				0.84				0.76				**0.02**				**<0.001**
Laddr use (≤12 h)	0.06	0.10	3,148	0.57	0.11	0.06	3,148	0.06	**0.31**	**0.07**	**3,148**	**<0.0001**	**−0.21**	**0.08**	**3,147**	**0.01**
No Laddr (≤12 h)	0.03	0.09	3,148	0.73	0.09	0.05	3,148	0.09	0.10	0.06	3,148	0.10	**0.19**	**0.07**	**3,147**	**0.01**
Caffeine				0.65				0.52				0.22				0.45
Laddr use (≤12 h)	0.08	0.05	3,148	0.09	−0.03	0.03	3,148	0.28	−0.05	0.03	3,148	0.16	−0.04	0.04	3,147	0.33
No Laddr (≤12 h)	0.05	0.05	3,148	0.24	−0.01	0.03	3,148	0.79	0.00	0.03	3,148	0.88	0.00	0.04	3,147	0.98
Likely smoke in next 4 h				0.69				0.19				0.28				0.83
Laddr use (≤12 h)	−0.02	0.02	3,148	0.33	0.03	0.01	3,148	0.006	0.01	0.01	3,148	0.68	0.04	0.02	3,147	0.02
No Laddr (≤12 h)	−0.01	0.02	3,148	0.69	0.01	0.01	3,148	0.28	−0.01	0.01	3,148	0.34	0.03	0.02	3,147	0.05
Temptation to smoke				0.87				0.70				**0.03**				**0.02**
Laddr use (≤12 h)	−0.03	0.02	3,148	0.07	0.01	0.01	3,148	0.30	**0.05**	**0.01**	**3,148**	**<0.0001**	**−0.05**	**0.01**	**3,147**	**<0.001**
No Laddr (≤12 h)	−0.04	0.02	3,148	0.03	0.02	0.01	3,148	0.09	0.01	0.01	3,148	0.18	−0.01	0.01	3,147	0.65
Urge to smoke				0.95				0.96				**0.004**				**0.03**
Laddr use (≤12 h)	−0.03	0.02	3,148	0.08	0.01	0.01	3,148	0.18	**0.05**	**0.01**	**3,148**	**<0.0001**	**−0.05**	**0.01**	**3,147**	**<0.001**
No Laddr (≤12 h)	−0.03	0.02	3,148	0.07	0.01	0.01	3,148	0.16	0.01	0.01	3,148	0.54	−0.01	0.01	3,147	0.45
Restraint in smoking				0.06				0.43				0.18				0.07
Laddr use (≤12 h)	0.05	0.01	3,148	<0.0001	−0.002	0.01	3,148	0.78	−0.02	0.01	3,148	0.002	0.03	0.01	3,147	0.001
No Laddr (≤12 h)	0.03	0.01	3,148	0.003	0.004	0.01	3,148	0.44	−0.01	0.01	3,148	0.08	0.01	0.01	3,147	0.22
Smoke since last prompt				0.19				0.13				0.33				0.33
Laddr use (≤12 h)	−0.07	0.08	3,148	0.37	0.09	0.04	3,148	0.04	−0.01	0.05	3,148	0.82	0.10	0.06	3,147	0.09
No Laddr (≤12 h)	0.07	0.08	3,148	0.39	0.00	0.05	3,148	1.00	0.06	0.05	3,148	0.29	0.02	0.06	3,147	0.71

*Parameter estimates from mixed-effects models with a context-by-Laddr interaction term*.

* *Regression coefficients presented in each row represent the association between the context measure and the momentary self-regulation outcome. For each context, they are presented for moments when Laddr had been used recently, and when Laddr had not been used recently. Bold values indicate p-values ≤ 0.05*.

### Associations Between Momentary Context Measures and Momentary Self-Regulation Subscales in the Binge-Eating Sample

#### Binge-Eating-Specific Context Measures

In the binge-eating sample, binge-related contexts were evaluated for their association with momentary self-regulation (Table 7). In this sample, a daily desire to binge eat was associated with lower momentary **perseverance** measures throughout the day. Momentary difficulty in accessing food and momentary restraint in eating were positively associated with momentary **perseverance**, while momentary urge to binge was negatively associated with momentary **perseverance**. These relationships are consistent with both external and internal context having an effect on an individual's perseverance. Higher values of daily uncontrolled and emotional eating (measured in the evening) were associated with lower momentary **perseverance** (measured throughout the day), while higher daily restrained eating was associated with higher momentary **perseverance**. A daily motivation to avoid binge eating (measured in the morning) was associated with a higher level of momentary **sensation seeking** (measured throughout the day). Momentary difficulty in accessing food was also associated with higher momentary **sensation seeking**. Daily desire to binge eat (measured in the morning), higher likelihood of binge eating in the next 4 h, momentary urge to binge eat, were all associated with higher levels of momentary **self-judgment**, while momentary restraint in eating was negatively associated with momentary **self-judgment**. Daily uncontrolled and emotional eating (measured in the evening) were also associated with momentary **self-judgment** (measured throughout the day). Taken together, these results indicate that self-judgement is engaged by contextual influence. Daily desire to binge eat (measured in the morning), momentary likelihood of binge eating in the next 4 h, and momentary urge to binge eat were all associated with lower momentary **mindfulness**, while momentary restraint in eating was associated with higher levels of momentary **mindfulness**. Daily uncontrolled, emotional, and restrained eating (measured in the evening) were also negatively associated with momentary **mindfulness**. These results indicate that binge-related contexts are associated with momentary self-regulation levels. Similar to results seen in the smoking sample, several contexts were associated with multiple aspects of momentary self-regulation: momentary perseverance, self-judgement, and mindfulness. Whereas only difficulty in accessing food was associated with momentary sensation seeking. Unlike in the smoking sample, the target risk behavior (binge-eating) was associated with all aspects of momentary self-regulation except sensation seeking.

**Table 7:** Momentary context measures, association with momentary self-regulation in the binge-eating sample.

	**Momentary perseverance**				**Momentary sensation seeking**				**Momentary self-judgment**				**Momentary mindfulness**			
	**β**	**SE**	**DF**	***p*-value**	**β**	**SE**	**DF**	***p*-value**	**β**	**SE**	**DF**	***p*-value**	**β**	**SE**	**DF**	***p*-value**
**Morning-only questions:**																
Desire to binge eat today	**−0.04**	**0.01**	**3,090**	**<0.0001**	−0.01	0.004	3,089	0.25	**0.02**	**0.01**	**3,090**	**<0.001**	**−0.04**	**0.01**	**3,090**	**<0.0001**
Motivated to avoid binge eating	0.01	0.01	3,090	0.28	**0.01**	**0.005**	**3,089**	**0.0478**	0.003	0.01	3,090	0.67	−0.002	0.01	3,090	0.78
**Questions on all prompts:**																
See or smell food	0.02	0.03	3,402	0.48	−0.003	0.02	3,401	0.88	0.02	0.03	3,402	0.46	−0.03	0.03	3,402	0.35
Hard to access food	**0.04**	**0.01**	**3,402**	**0.002**	**0.02**	**0.01**	**3,401**	**0.01**	0.01	0.01	3,402	0.46	**0.02**	**0.01**	**3,402**	**0.05**
Likely to binge eat in next 4 h	−0.03	0.02	3,401	0.07	0.01	0.01	3,400	0.52	**0.07**	**0.01**	**3,401**	**<0.0001**	**−0.11**	**0.01**	**3,401**	**<0.0001**
Urge to binge eat	**−0.06**	**0.01**	**3,402**	**<0.0001**	−0.003	0.01	3,401	0.75	**0.11**	**0.01**	**3,402**	**<0.0001**	**−0.10**	**0.01**	**3,402**	**<0.0001**
How hungry	0.02	0.01	3,402	0.13	−0.004	0.01	3,401	0.57	0.01	0.01	3,402	0.36	**−0.02**	**0.01**	**3,402**	**0.05**
Restraint in eating	**0.04**	**0.01**	**3,402**	**<0.0001**	−0.004	0.005	3,401	0.43	**−0.04**	**0.01**	**3,402**	**<0.0001**	**0.05**	**0.01**	**3,402**	**<0.0001**
Binge ate since last prompt	**−0.36**	**0.05**	**3,402**	**<0.0001**	0.02	0.03	3,401	0.56	**0.26**	**0.04**	**3,402**	**<0.0001**	**−0.27**	**0.04**	**3,402**	**<0.0001**
**Evening-only questions:**																
Uncontrolled eating	**−0.06**	**0.01**	**3,097**	**<0.0001**	0.001	0.01	3,096	0.88	**0.08**	**0.01**	**3,097**	**<0.0001**	**−0.10**	**0.01**	**3,097**	**<0.0001**
Emotional eating	**−0.04**	**0.01**	**3,097**	**0.005**	0.005	0.01	3,096	0.61	**0.07**	**0.01**	**3,097**	**<0.0001**	**−0.07**	**0.01**	**3,097**	**<0.0001**
Restrained eating	**0.06**	**0.02**	**3,097**	**<0.0001**	−0.01	0.01	3,096	0.26	0.02	0.01	3,097	0.08	**−0.03**	**0.01**	**3,097**	**0.02**

*Parameter estimates from mixed-effects models. Bold values indicate p-values ≤ 0.05*.

Recent Laddr use modified the association between some binge-related contexts and momentary self-regulation levels, as seen in the Laddr-by-context interaction models (Table 8). In particular, momentary urge to binge and momentary **perseverance**. When Laddr had *not* been used in the past 12 h, urge to binge was significantly associated with lower momentary perseverance, in contrast to a lack of significant association when Laddr had recently been used, possibly suggesting that use of Laddr decreased the influence of the internal context. Laddr use also modified the relationship between having binge eaten since the last prompt and momentary **perseverance**. The magnitude of the negative association was greater following Laddr use than it was in moments not following Laddr use, although in both cases the association was significant. It is unclear how to interpret this finding, but given that in this study Laddr included both EMA questions as well as the therapeutic intervention, reflecting on a recent binge episode as reported in EMA may reduce momentary perseverance. Recent Laddr use also modified the association between daily uncontrolled eating (measured in the evening) and momentary **perseverance** (measured throughout the day). The association was significant and negative following recent Laddr use but not significant when Laddr had not recently been used, possibly indicating a greater awareness of the link when Laddr was used. In contrast, for daily restraint in eating (measured in the evening), the positive association with momentary **perseverance** was only significant when Laddr had *not* recently been used. Again, the possible implication is unclear beyond the intervention's moderation of the association. Recent Laddr use modified the association between daily uncontrolled eating (measured in the evening) and momentary **sensation seeking**, with the direction of the association differing when Laddr had (negative) and had not (positive) been used. Specifically, when Laddr had been used, a higher daily level of uncontrolled eating was associated with lower momentary sensation seeking throughout the day, and when Laddr had not been used, a higher daily level of uncontrolled eating was associated with a higher level of momentary sensation seeking. The association between momentary hunger and momentary **self-judgment** also significantly differed by recent Laddr use; there was a significant positive association between momentary hunger and momentary **self-judgment** only when Laddr had not been used. A similar pattern was seen with daily desire to binge eat: only when Laddr had *not* been used recently was there a significant positive association between desire to binge and momentary **self-judgment**. Recent Laddr use also modified the association between daily desire to binge eat and momentary **mindfulness**, with only the magnitude of the significant negative association differing with and without recent Laddr use. As with the smoking sample, the binge-eating sample results likewise indicate the possibility that health behavior interventions are capable of modifying the influence of behavior-specific context on an individual's self-regulation in the moment.

**Table 8:** Momentary context measures, association with momentary self-regulation by recent Laddr use (≤12 h) in the binge-eating sample.

**Association between momentary context and momentary self-regulation measures** **Presented separately by recent Laddr use (≤12 h)**	**Momentary Perseverance**				**Momentary Sensation Seeking**				**Momentary Self-Judgment**				**Momentary Mindfulness**			
	β^*^	**SE**	**DF**	***p*-value**	β^*^	**SE**	**DF**	***p*-value**	β^*^	**SE**	**DF**	***p*-value**	β^*^	**SE**	**DF**	***p*-value**
**Morning-only questions:**																
Desire to binge eat today				0.10				0.07				**0.01**				**0.03**
Laddr use (≤12 h)	−0.03	0.01	3,089	0.002	−0.01	0.01	3,088	0.04	0.01	0.01	3,089	0.24	**−0.03**	**0.01**	**3,089**	**<0.0001**
No Laddr (≤12 h)	−0.05	0.01	3,089	<0.0001	0.001	0.01	3,088	0.84	**0.03**	**0.01**	**3,089**	**<0.0001**	**−0.05**	**0.01**	**3,089**	**<0.0001**
Motivated to avoid binge eating				0.76				0.31				0.14				0.21
Laddr use (≤12 h)	0.01	0.01	3,089	0.40	0.01	0.01	3,088	0.03	0.01	0.01	3,089	0.18	−0.01	0.01	3,089	0.33
No Laddr (≤12 h)	0.01	0.01	3,089	0.20	0.01	0.01	3,088	0.34	−0.004	0.01	3,089	0.62	0.01	0.01	3,089	0.54
**Questions on all prompts:**																
See or smell food				0.85				0.78				0.94				0.43
Laddr use (≤12 h)	0.03	0.05	3,401	0.52	−0.01	0.03	3,400	0.76	0.02	0.04	3,401	0.63	−0.01	0.04	3,401	0.87
No Laddr (≤12 h)	0.02	0.05	3,401	0.71	0.003	0.03	3,400	0.93	0.02	0.04	3,401	0.56	−0.05	0.04	3,401	0.22
Hard to access food				0.28				0.71				0.25				0.43
Laddr use (≤12 h)	0.03	0.02	3,401	0.09	0.02	0.01	3,400	0.11	0.02	0.02	3,401	0.19	0.02	0.02	3,401	0.33
No Laddr (≤12 h)	0.05	0.02	3,401	0.002	0.02	0.01	3,400	0.04	−0.003	0.02	3,401	0.84	0.03	0.02	3,401	0.04
Likely to binge eat in next 4 h				0.58				0.89				0.08				0.07
Laddr use (≤12 h)	−0.03	0.02	3,400	0.10	0.005	0.01	3,399	0.70	0.05	0.02	3,400	0.005	−0.13	0.02	3,400	<0.0001
No Laddr (≤12 h)	−0.05	0.02	3,400	0.02	0.01	0.01	3,399	0.58	0.09	0.02	3,400	<0.0001	−0.09	0.02	3,400	<0.0001
Urge to binge eat				**0.04**				0.33				0.36				0.17
Laddr use (≤12 h)	−0.03	0.02	3,401	0.08	−0.01	0.01	3,400	0.37	0.10	0.02	3,401	<0.0001	−0.12	0.02	3,401	<0.0001
No Laddr (≤12 h)	**−0.09**	**0.02**	**3,401**	**<0.0001**	0.005	0.01	3,400	0.67	0.12	0.02	3,401	<0.0001	−0.09	0.02	3,401	<0.0001
How hungry				0.87				0.85				**0.03**				0.92
Laddr use (≤12 h)	0.02	0.02	3,401	0.30	−0.003	0.01	3,400	0.77	−0.01	0.02	3,401	0.38	−0.02	0.02	3,401	0.13
No Laddr (≤12 h)	0.01	0.02	3,401	0.43	−0.01	0.01	3,400	0.58	**0.03**	**0.02**	**3,401**	**0.03**	−0.02	0.02	3,401	0.17
Restraint in eating				0.06				0.49				0.88				0.06
Laddr use (≤12 h)	0.04	0.01	3,401	<0.001	−0.01	0.01	3,400	0.30	−0.04	0.01	3,401	<0.0001	0.06	0.01	3,401	<0.0001
No Laddr (≤12 h)	0.06	0.01	3,401	<0.0001	−0.0007	0.01	3,400	0.91	−0.04	0.01	3,401	<0.0001	0.04	0.01	3,401	<0.0001
Binge ate				**0.004**				0.80				0.13				0.09
Laddr use (≤12 h)	**−0.49**	**0.07**	**3,401**	**<0.0001**	0.03	0.04	3,400	0.55	0.32	0.06	3,401	<0.0001	−0.34	0.06	3,401	<0.0001
No Laddr (≤12 h)	**−0.20**	**0.07**	**3,401**	**0.004**	0.01	0.04	3,400	0.81	0.19	0.06	3,401	0.002	−0.20	0.06	3,401	0.002
**Evening-only questions:**																
Uncontrolled eating				**0.01**				**<0.001**				0.83				0.10
Laddr use (≤12 h)	**−0.09**	**0.02**	**3,096**	**<0.0001**	**−0.03**	**0.01**	**3,095**	**0.03**	0.09	0.02	3,096	<0.0001	−0.12	0.02	3,096	<0.0001
No Laddr (≤12 h)	−0.02	0.02	3,096	0.33	**0.03**	**0.01**	**3,095**	**0.01**	0.08	0.02	3,096	<0.0001	−0.08	0.02	3,096	<0.0001
Emotional eating				0.86				0.82				0.12				0.74
Laddr use (≤12 h)	−0.05	0.02	3,096	0.01	0.01	0.01	3,095	0.58	0.05	0.02	3,096	0.004	−0.07	0.02	3,096	<0.0001
No Laddr (≤12 h)	−0.05	0.02	3,096	0.02	0.006	0.01	3,095	0.80	0.08	0.02	3,096	<0.0001	−0.08	0.02	3,096	<0.0001
Restrained eating				**0.001**				0.41				0.85				0.69
Laddr use (≤12 h)	0.02	0.02	3,096	0.32	−0.01	0.01	3,095	0.67	0.02	0.02	3,096	0.22	−0.03	0.02	3,096	0.09
No Laddr (≤12 h)	**0.10**	**0.02**	**3,096**	**<0.0001**	−0.02	0.01	3,095	0.16	0.03	0.02	3,096	0.15	−0.04	0.02	3,096	0.03

*Parameter estimates from mixed-effects models with a context-by-Laddr interaction term*.

* *Regression coefficients presented in each row represent the association between the context measure and the momentary self-regulation outcome. For each context, they are presented for moments when Laddr had been used recently, and when Laddr had not been used recently. Bold values indicate p-values ≤ 0.05*.

## Discussion

The goal of this study was to determine whether self-regulation varied within individual in different contexts among individuals who smoke and individuals who are overweight and have binge-eating disorder, two exemplar populations with health risk behaviors hypothesized to be related to self-regulation. It was not our aim in this study, to definitively identify the contextual drivers of self-regulation change within a smoking or within a binge-eating population. Rather, this study can be viewed as a first step to demonstrating that self-regulation is not static throughout the course of an individual's day, and that momentary self-regulation measures differ by both general and condition-specific contexts. It is also noteworthy that we were able to capture the fluctations in patterns of momentary self-regulation *via* the momentary self-regulation scale. The pattern of fluctuation by context was seen in two different populations, whose similarity is in the hypothesized underlying influence of self-regulation on the resultant risk behaviors characteristic of the condition. In addition, while evaluating the Laddr intervention was not the purpose of this study, this study was able to demonstrate that existing context-to-self-regulation relationships were modified when a self-regulation-based intervention was used (compared to when it was not). In this study, we were able to characterize samples from these populations at the trait level. We described their momentary levels of self-regulation and showed how these levels are associated with momentary contexts. Further, we were able to demonstrate that the Laddr mobile intervention modified some of these associations.

### Study Participants

The higher scores on the PHQ-8 in the binge-eating sample indicate that this population may have a higher burden of depression symptoms. This is consistent with the co-occurrence of binge-eating disorder and depression and other mental illnesses (62). The higher scores on several trait-level, baseline self-regulation measures in the smoking sample compared with the binge-eating sample could indicate that those who binge eat may have larger deficits in self-regulation on average than those who smoke. Alternatively, to the extent that worse self-regulation is related to greater severity of health risk behaviors, this finding may be at least partially explained by the eligibility criterion of BED in the binge-eating sample compared with the less severe criterion of smoking five or more tobacco cigarettes per day in the smoking sample.

### Associations With Momentary Self-Regulation

The ability to measure associations at the momentary level may help to elucidate the complex effects that an individual's context may have on their level of self-regulation and, in turn, their ability to limit health risk behaviors. Several general momentary contexts were associated with momentary self-regulation in the combined sample. Contexts that would be hypothesized to be more challenging, including higher stress level, greater level of tiredness, and negative affect, were associated with worse levels of momentary self-regulation—lower perseverance, higher self-judgment, and lower levels of mindfulness. Likewise, positive affect, which is hypothesized to have a positive rather than negative effect on self-regulation, was associated with higher momentary perseverance, lower levels of self-judgment, and higher levels of mindfulness. Together, these patterns suggest that momentary challenges and negative situations result in lower momentary levels of self-regulation, and conversely, it is in less challenging or positive situations that self-regulation is at its highest. Interestingly, challenging contexts, including negative affect and stress (but not tiredness), were associated with *lower* momentary sensation seeking, and the less challenging context of higher positive affect was associated with *higher* momentary sensation seeking. These findings indicate that the sensation-seeking component of momentary self-regulation may function differently than the other aspects of self-regulation with respect to the influence of more and less challenging momentary contexts, or perhaps that the definition of a challenging context may be somewhat different with respect to momentary sensation seeking as compared with momentary perseverance, self-judgment, and mindfulness.

Compared to being with others, being alone had varied associations with the momentary self-regulation subscales. Being alone, which is not clearly more or less challenging when it comes to self-regulation in general, was associated with lower momentary perseverance and lower momentary sensation seeking in the combined sample. This result may imply that situations where an individual is alone may be a potential target for self-regulation interventions focused on these facets of self-regulation.

Higher momentary temptation and urge to smoke were uniformly associated with worse momentary self-regulation in three subscales: lower perseverance, higher self-judgment, and lower mindfulness. Given these results in combination with previous findings that temptation and urge are associated with smoking likelihood (63, 64), future work should be conducted to investigate whether the associations of temptation and urge to smoke with smoking are mediated by momentary self-regulation. However, we did not see an association between momentary self-regulation and smoking in our sample. This may reflect the addiction component of smoking as a health risk behavior. That is, self-regulation may play a smaller role in influencing health risk behaviors when the behavior is more strongly driven by a physiological addiction. If a lack of association between momentary self-regulation and smoking is replicated in larger, more diverse samples, this finding would have implications for the effectiveness of smoking interventions focusing only on self regulation. We also did not see an association between temptation or urge to smoke with momentary sensation seeking. As with the smoking sample, momentary urge to binge eat—the health risk behavior—was associated with worse momentary self-regulation on three subscales: lower perseverance, higher self-judgment, and lower mindfulness, again suggesting that future studies should explore whether self-regulation is the mechanism by which urge and temptation influence health risk behaviors. The exception to this was that urge to binge eat was not associated with momentary sensation seeking. This may imply that sensation seeking plays a smaller role than the other aspects of momentary self-regulation in binge eating. Further, having binge eaten since the last prompt was associated with worse momentary self-regulation in terms of lower perseverance, higher self-judgment, and lower mindfulness, but there was no association with sensation seeking. In contrast, having smoked since the last prompt was not associated with any of the momentary self-regulation subscales, which may be explained by the smoking sample's relatively low desire to avoid smoking on a daily basis. Lapses in self-regulation are predicated on a desire to avoid the health risk behavior. It is perhaps unsurprising that a group unmotivated to refrain from smoking would have smoking episodes that are not associated with momentary self-regulation.

Difficulty accessing cigarettes in the smoking sample and difficulty accessing food in the binge-eating sample were each associated with a higher level of momentary perseverance. This may reflect that limiting access to cigarettes or food in the smoking and binge-eating samples, respectively, results in better self-regulation. If so, reducing access may be a strategy for controlling the health risk behavior. It is possible that those best at self-regulating may do so not primarily through the exercise of willpower in individual events but instead by modifying their environments to limit their risk exposures.

Several smoking cues, including seeing others smoke, smelling smoke, and drinking alcohol, were associated with higher sensation seeking; future work should test the hypothesis that exposure to smoking cues increases the likelihood of smoking *via* higher sensation seeking. Drinking alcohol and smelling smoke were associated with higher levels of momentary self-judgment, but it is unclear if this higher self-judgment was in response to the smoking cue or the fact that exposure to the smoking cue resulted in the individual smoking. We did not collect information about whether the smoking cue exposure occurred before or after the reported smoking episode. To the extent that they reflect cue exposures before smoking, these findings align with previous research on the role of smoking cues with respect to craving and smoking (65–67) and add evidence in real-world rather than laboratory-based contexts, where results may not be consistent (68). In the binge-eating sample, seeing or smelling food and momentary hunger level were not associated with any of the momentary self-regulation subscales. These results add to the conflicting literature on cues and binge eating, though the lack of associations may be due in part to cue exposures both before and after binge-eating episodes. A laboratory-based study found that food cues increase food craving in individuals with binge-eating disorder or bulimia nervosa (69), but a qualitative interview study of women found that binge-eating episodes were not strongly driven by food cues (70). In contrast to our finding of no association, a previous EMA study of adults with obesity found that relative to non-binge-eating episodes, binge-eating episodes were associated with lower pre-episode hunger (71). The role of cues in smoking and binge eating should be further explored in real-world contexts.

While there was not a direct association between recent Laddr use and the momentary self-regulation subscales in the combined sample, or the smoking and binge-eating samples, recent Laddr use modified the relationship between some of the momentary contexts and momentary self-regulation subscales. In particular, in the combined as well as the smoking and binge-eating samples, some associations between location and momentary self-regulation subscales differed by whether Laddr was recently used. Also, in both the smoking sample and the binge-eating sample, recent Laddr use appeared to modify some of the relationships between challenging contexts and momentary self-regulation. These findings demonstrate the possibility of measuring the effects of momentary, mobile interventions in context and at a momentary level.

### Limitations

The study had several limitations. This was not a nationally representative sample of the US; it had limited racial and ethnic diversity and had a higher proportion of individuals with advanced education than would be expected in a more representative sample. Although recruitment was conducted nationally, recruitment was also conducted in a rural, predominately white area with both an academic medical center and a large college. This likely influenced the distribution of participants recruited for the study. Differences between the smoking and binge-eating samples may reflect differences in the general populations of individuals who smoke and individuals who are overweight and have binge-eating disorder; however, the distribution of some demographics indicates that the study sample may lack generalizability to these populations in the US. For example, while binge-eating disorder has a higher prevalence among females (72), smoking has a slightly higher prevalence among males in the US (73, 74). The predominance of women in both samples is therefore most likely due to unintended sampling and/or self-selection bias. Also, the inclusion criteria were developed to create non-overlapping samples, so results may not generalize to the population of individuals who smoke and have binge-eating disorder. Additionally, measures of smoking and binge eating were self-reported and were not validated with breath samples or objective measures of eating. Finally, the study was exploratory in nature, and many associations were tested, allowing for the possibility of spurious associations. With a 0.05 significance level, by chance, we would expect about 2 significant results among the 40 tests of association with momentary contexts in the combined sample, even if no true associations existed; in the sample, 23 reached statistical significance. Similarly, even with no true effects, we would expect about 2–3 significant results among the 56 tests when evaluating smoking-specific contexts in the smoking sample; in the smoking sample, 20 reached statistical significance. With no true effect, we would expect about 2–3 significant results among the 48 tests when evaluating binge-eating-specific contexts in the binge-eating sample; in the binge-eating sample 27 tests reached statistical significance. The same is true when the 40, 56 and 48 sample-specific context-by-Laddr interaction tests were performed, respectively, where 1, 10, and 8 tests reached statistical significance in the sample. The associations were not controlled for potential confounders, and the effect sizes for many of the associations are relatively small. Future studies are needed to confirm the initial associations identified in the current work.

### Conclusions

Overall, this study provides preliminary evidence that momentary self-regulation may vary in response to differing internal and external contexts in samples from two exemplar populations with health risk behaviors. In addition, the Laddr intervention appears to modify some of these relationships. Therefore, the influence of contextual factors on health risk behaviors may occur *via* momentary influences on self-regulation. Future studies of self-regulation should consider measuring momentary self-regulation in similar and different samples to determine the replicability of the findings, or further explore particular types of contexts. If the results replicate, future health behavior change interventions may wish to consider measuring and targeting momentary self-regulation in addition to trait-level self-regulation to better understand and improve health risk behaviors. In the context of the larger SOBC project, this work will be used to inform a later stage of research focused on assessing the transdiagnostic mediating effect of momentary self-regulation on medical regimen adherence and health outcomes.

## Data Availability Statement

The raw data supporting the conclusions of this article will be made available by the authors, without undue reservation.

## Ethics Statement

The studies involving human participants were reviewed and approved by Dartmouth College Institutional Review Board. The patients/participants provided their written informed consent to participate in this study.

## Author Contributions

RP, DM, and LM conceived the study and obtained research funding. ES, SM, CW, SK, MS, HX, PB, JD, MK, ML, LO, JP, LS, RP, DM, and LM contributed to the study's design. SM, CW, and LM wrote the protocol. SM, CW, MG, and MS implemented the data collection instruments. SM, CW, SB, and HL recruited and enrolled participants and collected the data. ES, SM, CW, SB, MG, and SL contributed to data monitoring and management. MG created, updated, and administered the mobile application and processed the momentary data for analysis. ES performed the statistical analyses and drafted the manuscript. SM and LM wrote sections of the manuscript. All authors contributed to manuscript revision and have approved the final version.

## Funding

This research was supported by the National Institutes of Health (NIH) Science of Behavior Change (SOBC) Common Fund through an award administered by the National Institute on Drug Abuse (Grant UH2DA041713).

## Author Disclaimer

Any opinions, findings, conclusions, or recommendations expressed in this material are those of the authors and do not necessarily reflect the views of the National Institute on Aging or the National Institutes of Health.

## Conflict of Interest

JP has provided consultation to pharmaceutical and technology companies that make medications and other treatments for quitting smoking and has served as an expert witness in lawsuits against tobacco companies. In addition to her academic affiliation, LM is affiliated with Square2 Systems, Inc., the small business that developed the mobile Laddr intervention and has worked extensively with relevant institutions (including Dartmouth College and NIH) to manage any potential conflict of interest. All research data collection, data management, and statistical analyses are conducted by individuals with no affiliation with Square2 Systems. The remaining authors declare that the research was conducted in the absence of any commercial or financial relationships that could be construed as a potential conflict of interest.

## Publisher's Note

All claims expressed in this article are solely those of the authors and do not necessarily represent those of their affiliated organizations, or those of the publisher, the editors and the reviewers. Any product that may be evaluated in this article, or claim that may be made by its manufacturer, is not guaranteed or endorsed by the publisher.

## References

[1] Eisenberg IW Bissett PG Canning JR Dallery J Enkavi AZ Whitfield-Gabrieli S. Applying novel technologies and methods to inform the ontology of self-regulation. Behav Res Therapy. (2018) 101:46–57. 10.1016/j.brat.2017.09.014 29066077 PMC5801197

[2] Bickel WK Odum AL Madden GJ. Impulsivity and cigarette smoking: Delay discounting in current, never, and ex-smokers. Psychopharmacology. (1999) 146:447–54. 10.1007/PL00005490 10550495

[3] Alessi SM Petry NM. Pathological gambling severity is associated with impulsivity in a delay discounting procedure. Behavioural Processes. (2003) 64:345–54. 10.1016/S0376-6357(03)00150-5 14580703

[4] Epstein LH Salvy SJ Carr KA Dearing KK Bickel WK. Food reinforcement, delay discounting and obesity. Physiol Behav. (2010) 100:438–5. 10.1016/j.physbeh.2010.04.029 20435052 PMC9632539

[5] de Ridder DTD de Wit JBF. Self-Regulation in Health Behavior. London: John Wiley and S (2008).

[6] Dingemans A Danner U Parks M. Emotion regulation in binge eating disorder: a review. Nutrients. (2017) 9:11. 10.3390/nu9111274 29165348 PMC5707746

[7] Bickel WK Marsch LA. A future for the prevention and treatment of drug abuse: Applications of computer-based interactive technology, In: Henningfield JE Santora PB Bickel WK, editors. Addiction treatment: Science and policy for the twenty-first century. Baltimore, MD: Johns Hopkins University Pr. (2007) p. 35–43.

[8] Abraham C Norman P Conner M. Understanding and Changing Health Behaviour: From Health Beliefs to Self-Regulation. Understanding and Changing Health Behaviour. London: Psychology Pr (2014).

[9] Roos CR Witkiewitz K. A contextual model of self-regulation change mechanisms among individuals with addictive disorders. Clinic Psychol Rev. (2017) 57:117–28. 10.1016/j.cpr.2017.08.008 28866435 PMC6152904

[10] Geisler FCM Kubiak T Siewert K Weber H. Cardiac vagal tone is associated with social engagement and self-regulation. Biol Psychol. (2013) 93:13. 10.1016/j.biopsycho.2013.02.013 23466587

[11] Roos CR Kober H Trull TJ MacLean RR Mun CJ. Intensive longitudinal methods for studying the role of self-regulation strategies in substance use behavior change. Curr Addict Rep. (2020) 7:5. 10.1007/s40429-020-00329-5 33510995 PMC7837607

[12] Gross JJ. The extended process model of emotion regulation: elaborations, applications, and future directions. Psychologic Inq. (2015) 26:511. 10.1080/1047840X.2015.989751

[13] Gross JJ. Antecedent- and response-focused emotion regulation: divergent consequences for experience, expression, and physiology. J Personal Soc Psychol. (1998) 74:224. 10.1037/0022-3514.74.1.224 9457784

[14] Schmeichel BJ. Attention control, memory updating, and emotion regulation temporarily reduce the capacity for executive control. J Experiment Psychol General. (2007) 136:241. 10.1037/0096-3445.136.2.241 17500649

[15] Sheppes G Scheibe S Suri G Gross JJ. Emotion-regulation choice. Psychologic Sci. (2011) 22:11. 10.1177/0956797611418350 21960251

[16] Dunton GF Rothman AJ Leventhal AM Intille SS. How intensive longitudinal data can stimulate advances in health behavior maintenance theories and interventions. Translat Behav Med. (2021) 11:281–6. 10.1093/tbm/ibz165 31731290 PMC7877301

[17] Marsch LA. Opportunities and needs in digital phenotyping. Neuropsychopharmacology. (2018) 43:1637–8. 10.1038/s41386-018-0051-7 29703995 PMC6006437

[18] Schroeder SA. We can do better—improving the health of the American people. New Engl J Med. (2007) 357:1221–8. 10.1056/NEJMsa073350 17881753

[19] Shiffman S Stone AA Hufford MR. Ecological momentary assessment. Ann Rev Clinic Psychol. (2008) 4:1–32. 10.1146/annurev.clinpsy.3.022806.091415 18509902

[20] Asch DA Muller RW Volpp KG. Automated hovering in health care — Watching over the 5000 h. New Engl J Med. (2012) 367:1–3. 10.1056/NEJMp1203869 22716935

[21] Sumner JA Carey RN Michie S Johnston M Edmondson D Davidson KW. Using rigorous methods to advance behaviour change science. Nat Hum Behav. (2018) 2:11. 10.1038/s41562-018-0471-8 30931398 PMC6437667

[22] Centers for Disease Control and Prevention (US); National Center for Chronic Disease Prevention and Health Promotion (US); Office on Smoking and Health (US). Nicotine addiction: Past and present, In: *How Tobacco Smoke Causes Disease: The Biology and Behavioral Basis for Smoking-Attributable Disease: A Report of the Surgeon General*. Georgia, AL: Centers for Disease Control and Prevention (US); (2010). 21452462

[23] Gearhardt AN White MA Potenza MN. Binge eating disorder and food addiction. Curr Drug Abuse Rev. (2011) 4:201–7. 10.2174/1874473711104030201 21999695 PMC3671377

[24] Hauck C Cook B Ellrott T. Food addiction, eating addiction and eating disorders. Proceed Nutri Soc. (2020) 79:103–12. 10.1017/S0029665119001162 31744566

[25] Hilbert A. Binge-eating disorder. Psychiatric Clinics N Am. (2019) 42:33–43. 10.1016/j.psc.2018.10.011 30704638

[26] Square2 Systems Inc. Laddr. Hanover, New Hampshire (2017).

[27] Yanovski SZ Marcus MD Wadden TA Walsh BT. The questionnaire on eating and weight patterns-5: an updated screening instrument for binge eating disorder. Int J Eat Disord. (2015) 48:259–61. 10.1002/eat.22372 25545458 PMC4374019

[28] American Psychiatric Association. Feeding and eating disorders, In: Diagnostic and Statistical Manual of Mental Disorders. 5th ed. Arlington, Virginia: American Psychiatric Associat (2013).

[29] Harris PA Taylor R Thielke R Payne J Gonzalez N Conde JG. Research electronic data capture (REDCap)—A metadata-driven methodology and workflow process for providing translational research informatics support. J Biomed Inform. (2009) 42:377–81. 10.1016/j.jbi.2008.08.010 18929686 PMC2700030

[30] Harris PA Taylor R Minor BL Elliott V Fernandez M O'Neal L. The REDCap consortium: Building an international community of software platform partners. J Biomed Inform. (2019) 95:209. 10.1016/j.jbi.2019.103208 31078660 PMC7254481

[31] Torrey WC Cepeda M Castro S Bartels SM Cubillos L Obando FS. Implementing technology-supported care for depression and alcohol use disorder in primary care in Colombia: Preliminary findings. Psychiatric Serv. (2020) 71:678–83. 10.1176/appi.ps.201900457 32151216 PMC7332379

[32] Marsch LA. Sector perspective: Digital therapeutics in behavioral health, In: van Dam J Sverdlov A, editors. Digital Therapeutics: Scientific, Statistical, Clinical, and Regulatory Aspects. CRC Pr In press.

[33] Acosta MC Possemato K Maisto SA Marsch LA Barrie K Lantinga L. Web-delivered CBT reduces heavy drinking in OEF-OIF veterans in primary care with symptomatic substance use and PTSD. Behav Therapy. (2017) 48:262–76. 10.1016/j.beth.2016.09.001 28270335 PMC5345259

[34] Dallery J Raiff BR Kim SJ Marsch LA Stitzer M Grabinski MJ. Nationwide access to an internet-based contingency management intervention to promote smoking cessation: A randomized controlled trial. Addiction. (2017) 112:875–83. 10.1111/add.13715 27923264 PMC5382065

[35] Marsch LA Gómez-Restrepo C Bartels SM Bell K Camblor PM Castro S. Scaling up science-based care for depression and unhealthy alcohol use in Colombia: an implementation science project. Psychiatric Serv. (2021) 21:202. 10.1176/appi.ps.202000041 34347504 PMC8810677

[36] Kroenke K Strine TW Spitzer RL Williams JBW Berry JT Mokdad AH. The PHQ-8 as a measure of current depression in the general population. J Affect Disord. (2009) 114:163–73. 10.1016/j.jad.2008.06.026 18752852

[37] Mason AE Vainik U Acree M Tomiyama AJ Dagher A Epel ES. Improving assessment of the spectrum of reward-related eating: the RED-13. Front Psychol. (2017) 8:795. 10.3389/fpsyg.2017.00795 28611698 PMC5447741

[38] Heatherton TF Kozlowski LT Frecker RC Fagerström K-O. The Fagerström test for nicotine dependence: a revision of the fagerstrom tolerance questionnaire. British Journal of Addiction. (1991) 86:1119–27. 10.1111/j.1360-0443.1991.tb01879.x 1932883

[39] Carey KB Neal DJ Collins SE. A psychometric analysis of the self-regulation questionnaire. Addict Behav. (2004) 29:253–60. 10.1016/j.addbeh.2003.08.001 14732414

[40] Gross JJ John OP. Individual differences in two emotion regulation processes: Implications for affect, relationships, and well-being. J Personal Soc Psychol. (2003) 85:348–62. 10.1037/0022-3514.85.2.348 12916575

[41] Baer RA Smith GT Hopkins J Krietemeyer J Toney L. Using self-report assessment methods to explore facets of mindfulness. Assessment. (2006) 13:27–45. 10.1177/1073191105283504 16443717

[42] Brown KW Ryan RM. The benefits of being present: Mindfulness and its role in psychological well-being. J Personal Soc Psychol Am Psychol. (2003) 24:822–48. 10.1037/0022-3514.84.4.822 12703651

[43] Baltes PB Baltes MM Freund AM Lang FR. The measure of selection, optimization, and compensation (SOC) by self-report. Berlin. (1999) 25:48. 10.1037/t04704-00011999929

[44] Whiteside SP Lynam DR. The Five Factor Model and impulsivity: Using a structural model of personality to understand impulsivity. Personal Individ Differen. (2001) 30:669–89. 10.1016/S0191-8869(00)00064-7

[45] Whiteside SP Lynam DR Miller JD Reynolds SK. Validation of the UPPS impulsive behaviour scale: A four-factor model of impulsivity. Euro J Personal. (2005) 19:559–74. 10.1002/per.55628118729

[46] Lynam DR Smith GT Whiteside SP Cyders MA. The UPPS-P: Assessing five personality pathways to impulsive behavior. West Lafayette, Indiana: Purdue Univers. (2006)

[47] Eysenck SBG Pearson PR Easting G Allsopp JF. Age norms for impulsiveness, venturesomeness and empathy in adults. Personal Individ Differen. (1985) 6:613–9. 10.1016/0191-8869(85)90011-X

[48] Shiffman S Kirchner TR Ferguson SG Scharf DM. Patterns of intermittent smoking: an analysis using ecological momentary assessment. Addict Behav. (2009) 34:514–9. 10.1016/j.addbeh.2009.01.004 19232834 PMC2855372

[49] Shiffman S Dunbar MS Li X Scholl SM Tindle HA Anderson SJ. Smoking patterns and stimulus control in intermittent and daily smokers. PLoS ONE. (2014) 9:3. 10.1371/journal.pone.0089911 24599056 PMC3943840

[50] Shrier LA Walls CE Kendall AD Blood EA. The context of desire to use marijuana: Momentary assessment of young people who frequently use marijuana. Psychol Addict Behav. (2012) 26:821–9. 10.1037/a0029197 22823544

[51] Shrier LA Scherer EB. It depends on when you ask: Motives for using marijuana assessed before vs. after a marijuana use event. Addict Behav. (2014) 39:1759–65. 10.1016/j.addbeh.2014.07.018 25123342 PMC4164570

[52] Wilhelm P Schoebi D. Assessing mood in daily life: Structural validity, sensitivity to change, and reliability of a short-scale to measure three basic dimensions of mood. Euro J Psychologic Assess. (2007) 23:258–67. 10.1027/1015-5759.23.4.258

[53] Hedeker D Mermelstein RJ Berbaum ML Campbell RT. Modeling mood variation associated with smoking: An application of a heterogeneous mixed-effects model for analysis of ecological momentary assessment (EMA) data. Addiction. (2009) 104:297–307. 10.1111/j.1360-0443.2008.02435.x 19149827 PMC2629640

[54] Goldschmidt AB Crosby RD Cao L Engel SG Durkin N Beach HM. Ecological momentary assessment of eating episodes in obese adults. Psychosomatic Med. (2014) 76:747–52. 10.1097/PSY.0000000000000108 25373891 PMC4530605

[55] Bond DS Thomas JG Ryder BA Vithiananthan S Pohl D Wing RR. Ecological momentary assessment of the relationship between intention and physical activity behavior in bariatric surgery patients. Int J Behav Med. (2013) 20:82–7. 10.1007/s12529-011-9214-1 22203518 PMC3909966

[56] Fitzsimmons-Craft EE Ciao AC Accurso EC. A naturalistic examination of social comparisons and disordered eating thoughts, urges, and behaviors in college women. Int J Eat Disord. (2016) 49:143–52. 10.1002/eat.22486 26610301 PMC4733430

[57] Stein RI Kenardy J Wiseman CV Dounchis JZ Arnow BA Wilfley DE. What's driving the binge in binge eating disorder?: A prospective examination of precursors and consequences. Int J Eat Disord. (2007) 40:195–203. 10.1002/eat.20352 17103418

[58] Thomas JG Doshi S Crosby RD Lowe MR. Ecological momentary assessment of obesogenic eating behavior: Combining person-specific and environmental predictors. Obesity. (2011) 19:1574–9. 10.1038/oby.2010.335 21273995

[59] Watson D Clark LA. The PANAS-X: Manual for the positive and negative affect schedule—expanded form. Ames I. (1994) 94:2. 10.17077/48vt-m4t2

[60] Karlsson J Persson LO Sjöström L Sullivan M. Psychometric properties and factor structure of the Three-Factor Eating Questionnaire (TFEQ) in obese men and women. Results from the Swedish Obese Subjects (SOS) study. Int J Obesity Relat Metabol Disorders : J Int Assoc Study Obesity. (2000) 24:1715–25. 10.1038/sj.ijo.0801442 11126230

[61] SAS Institute Inc. SAS software v9.4. Cary, North Carolina (2013).

[62] Javaras KN Pope HG Lalonde JK Roberts JL Nillni YI Laird NM. Co-occurrence of binge eating disorder with psychiatric and medical disorders. J Clinic Psychiatr. (2008) 69:266–73. 10.4088/JCP.v69n0213 18348600

[63] Cambron C Haslam AK Baucom BRW Lam C Vinci C Cinciripini P. Momentary precipitants connecting stress and smoking lapse during a quit attempt. Health Psychol. (2019) 38:1049–58. 10.1037/hea0000797 31556660 PMC6861642

[64] Shmueli D Prochaska JJ. Resisting tempting foods and smoking behavior: Implications from a self-control theory perspective. Health Psychol. (2009) 28:300–6. 10.1037/a0013826 19450035 PMC2736876

[65] Conklin CA Vella EJ Joyce CJ Salkeld RP Perkins KA Parzynski CS. Examining the relationship between cue-induced craving and actual smoking. Experiment Clinic Psychopharmacol. (2015) 23:90–6. 10.1037/a0038826 25730416 PMC4412312

[66] Michalowski A Erblich J. Reward dependence moderates smoking-cue- and stress-induced cigarette cravings. Addict Behav. (2014) 39:1879–83. 10.1016/j.addbeh.2014.07.032 25133977 PMC4164590

[67] Balter LJT Good KP Barrett SP. Smoking cue reactivity in current smokers, former smokers and never smokers. Addict Behav. (2015) 45:26–9. 10.1016/j.addbeh.2015.01.010 25635692

[68] Shiffman S Li X Dunbar MS Tindle HA Scholl SM Ferguson SG. Does laboratory cue reactivity correlate with real-world craving and smoking responses to cues? Drug and Alcohol Depend. (2015) 155:163–9. 10.1016/j.drugalcdep.2015.07.673 26277429 PMC4581999

[69] Meule A Küppers C Harms L Friederich HC Schmidt U Blechert J. Food cue-induced craving in individuals with bulimia nervosa and binge-eating disorder. PLoS ONE. (2018) 13:9. 10.1371/journal.pone.0204151 30212574 PMC6136823

[70] Bohon C Matheson B Welch H. Descriptive analysis of binge eating in adult and adolescent females. Eating Weight Disord. (2021) 26:1149–58. 10.1007/s40519-020-01013-3 32960440

[71] Goldschmidt AB Crosby RD Cao L Wonderlich SA Mitchell JE Engel SG. A preliminary study of momentary, naturalistic indicators of binge-eating episodes in adults with obesity. Int J Eat Disord. (2018) 51:87–91. 10.1002/eat.22795 29112288 PMC5745052

[72] Hudson JI Hiripi E Pope HG Kessler RC. The prevalence and correlates of eating disorders in the National Comorbidity Survey Replication. Biol Psychiatry. (2007) 61:348–58. 10.1016/j.biopsych.2006.03.040 16815322 PMC1892232

[73] Higgins ST Kurti AN Redner R White TJ Gaalema DE Roberts ME. A literature review on prevalence of gender differences and intersections with other vulnerabilities to tobacco use in the United States, 2004-2014. Prevent Med. (2015) 80:89–100. 10.1016/j.ypmed.2015.06.009 26123717 PMC4592404

[74] Jamal A King BA Neff LJ Whitmill J Babb SD Graffunder CM. Current cigarette smoking among adults—United States, 2005–2015. MMWR Morbidity Mortality Weekly Rep. (2016) 65:1205–11. 10.15585/mmwr.mm6544a2 27832052

