# Supplementary material for: Momentary Influences on Self-Regulation in Two Populations With Health Risk Behaviors: Adults Who Smoke and Adults Who Are Overweight and Have Binge-Eating Disorder
Source: Front Digit Health. 2022 Mar 18;4:798895. doi: 10.3389/fdgth.2022.798895 (PMC8971561; doi:10.3389/fdgth.2022.798895)
Supplement: Supplementary file 1 [file Data_Sheet_1.docx]

**Supplemental Tables**

Supplemental Table 1. Momentary self-regulation and context measures; p-value from generalized estimating equations to evaluate differences between samples.

|  | **Combined sample**  **N=159 participants**  **6714 observations** | | | **Smoking sample**  **N=82 participants**  **3233 observations** | | | **Binge-eating sample**  **N=77 participants**  **3481 observations** | | | **p-value** |
| --- | --- | --- | --- | --- | --- | --- | --- | --- | --- | --- |
|  | N | Mean  % | SD | N | Mean  % | SD | N | Mean  % | SD |  |
| Momentary self-regulation |  |  |  |  |  |  |  |  |  |  |
| Perseverance | 6714 | 3.0 | 1.2 | 3233 | 3.0 | 1.2 | 3481 | 3.1 | 1.2 | 0.29 |
| Sensation seeking | 6713 | 2.2 | 0.8 | 3233 | 2.2 | 0.8 | 3480 | 2.1 | 0.8 | 0.95 |
| Self-judgment | 6714 | 1.9 | 1.1 | 3233 | 1.6 | 0.9 | 3481 | 2.2 | 1.1 | <.001 |
| Mindfulness | 6713 | 3.9 | 1.1 | 3232 | 4.2 | 1.0 | 3481 | 3.6 | 1.1 | <.001 |
| At this moment are you: |  |  |  |  |  |  |  |  |  | 0.93 |
| With others | 3771 | 56.2% |  | 1770 | 54.7% |  | 2001 | 57.5% |  |  |
| Alone | 2943 | 43.8% |  | 1463 | 45.3% |  | 1480 | 42.5% |  |  |
| At this moment, where are you: |  |  |  |  |  |  |  |  |  | <.001 |
| Home  (own or friend’s or family member’s) | 3913 | 58.3% |  | 2175 | 67.3% |  | 1738 | 49.9% |  |  |
| Car | 613 | 9.1% |  | 288 | 8.9% |  | 325 | 9.3% |  |  |
| Work | 1439 | 21.4% |  | 587 | 18.2% |  | 852 | 24.5% |  |  |
| Other  (school, cafeteria/restaurant/bar, outside, other) | 749 | 11.2% |  | 183 | 5.7% |  | 566 | 16.3% |  |  |
| Right now: positive affect (1: not at all – 7: extremely) | 6714 | 3.8 | 1.3 | 3233 | 3.9 | 1.3 | 3481 | 3.8 | 1.3 | 0.63 |
| Right now: negative affect (1: not at all – 7: extremely) | 6714 | 2.5 | 1.4 | 3233 | 2.4 | 1.4 | 3481 | 2.6 | 1.3 | 0.11 |
| Right now: how stressed do you feel? (1: not at all – 7: extremely) | 6714 | 3.3 | 1.9 | 3233 | 3.2 | 2.0 | 3481 | 3.5 | 1.8 | 0.22 |
| Right now: how tired do you feel? (1: not at all – 7: extremely) | 6714 | 4.1 | 1.8 | 3233 | 3.8 | 1.8 | 3481 | 4.3 | 1.7 | 0.02 |
| Used Laddr (≤12 hrs) | 3223 | 48.0% |  | 1470 | 45.5% |  | 1753 | 50.4% |  | 0.37 |
| Used Laddr (≤1 hr) | 751 | 11.2% |  | 379 | 11.7% |  | 372 | 10.7% |  | 0.35 |

Supplemental Table 2. Momentary context measures in the **smoking sample**

|  | N | Mean  % | SD |
| --- | --- | --- | --- |
| **Morning-only questions:** |  |  |  |
| Desire to smoke today (0: no desire – 5: neutral – 10: very strong desire) | 793 | 6.2 | 2.2 |
| How motivated to avoid smoking today (0: not at all – 5: neutral – 10: very strongly) | 793 | 4.3 | 2.7 |
| **Questions on all prompts:** |  |  |  |
| Seen other smoke | 1196 | 37.0% |  |
| Seen cigarette | 2748 | 85.0% |  |
| Smelled smoke | 1342 | 44.5% |  |
| How hard to access cigarettes (1: very easy – 5: very hard) | 3233 | 1.4 | 1.0 |
| Eaten | 1944 | 60.1% |  |
| Alcohol | 169 | 5.2% |  |
| Caffeine | 1768 | 54.7% |  |
| How likely smoke in next 4 hours (1: very unlikely – 5: very likely) | 3233 | 4.2 | 1.2 |
| Temptation to smoke (1: not at all – 5: very much) | 3233 | 2.8 | 1.4 |
| Urge to smoke (1: not at all – 5: very much) | 3233 | 2.9 | 1.4 |
| Restraint in smoking (1: no restraint – 10: total restraint) | 3233 | 5.0 | 2.6 |
| Smoked since last prompt | 2891 | 89.4% |  |

Supplemental Table 3. Momentary context measures in the **binge-eating sample**

|  | N | Mean  % | SD |
| --- | --- | --- | --- |
| **Morning-only questions:** |  |  |  |
| Desire to binge eat today (0: no desire – 5: neutral – 10: very strong desire) | 865 | 3.5 | 2.8 |
| How motivated to avoid binge eating today (0: not at all – 5: neutral – 10: very strongly) | 865 | 7.0 | 2.5 |
| **Questions on all prompts:** |  |  |  |
| See or smell food | 2309 | 66.3% |  |
| How hard to access food (1: very easy – 5: very hard) | 3481 | 1.9 | 1.3 |
| How likely to binge eat in next 4 hours (1: very unlikely – 5: very likely) | 3480 | 2.0 | 1.1 |
| Urge to binge eat (1: not at all – 5: extremely) | 3481 | 2.0 | 1.2 |
| How hungry (1: not at all hungry – 5: extremely hungry) | 3481 | 2.4 | 1.2 |
| Restraint in eating (1: no restraint – 10: total restraint) | 3481 | 6.8 | 2.2 |
| Binge ate | 350 | 10.1% |  |
| **Evening-only questions:** |  |  |  |
| Couldn’t stop eating (1: not at all true for me – 5: very true for me) | 867 | 2.3 | 1.3 |
| Consoled myself when negative emotions (1–5) | 867 | 2.5 | 1.3 |
| Constantly limited intake/avoided foods (1–5) | 867 | 2.2 | 1.3 |

Supplemental Table 4. Baseline measures, association with momentary self-regulation in the **combined sample**. Parameter estimates from mixed-effects models.

|  | **Momentary Perseverance** | | | | **Momentary Sensation Seeking** | | | | **Momentary Self-Judgment** | | | | **Momentary Mindfulness** | | | |
| --- | --- | --- | --- | --- | --- | --- | --- | --- | --- | --- | --- | --- | --- | --- | --- | --- |
| **Baseline measure** | β | SE | DF | p-value | β | SE | DF | p-value | β | SE | DF | p-value | β | SE | DF | p-value |
| RED-13 | -0.00007 | 0.01 | 6554 | 0.99 | -0.004 | 0.003 | 6553 | 0.21 | 0.02 | 0.005 | 6554 | <.0001 | -0.03 | 0.004 | 6553 | <.0001 |
| SSRQ | 0.01 | 0.004 | 6554 | <.001 | 0.01 | 0.003 | 6553 | 0.01 | -0.02 | 0.004 | 6554 | <.0001 | 0.02 | 0.004 | 6553 | <.0001 |
| ERQ cognitive reappraisal | 0.03 | 0.01 | 6554 | 0.005 | 0.01 | 0.01 | 6553 | 0.02 | -0.03 | 0.01 | 6554 | 0.009 | 0.03 | 0.01 | 6553 | 0.003 |
| ERQ expressive suppression | -0.02 | 0.01 | 6554 | 0.23 | 0.004 | 0.01 | 6553 | 0.63 | 0.03 | 0.01 | 6554 | 0.02 | -0.01 | 0.01 | 6553 | 0.31 |
| FFMQ observing | 0.03 | 0.01 | 6553 | 0.01 | 0.01 | 0.01 | 6553 | 0.39 | -0.02 | 0.01 | 6553 | 0.15 | 0.03 | 0.01 | 6553 | 0.01 |
| FFMQ describing | 0.04 | 0.01 | 6554 | <.0001 | 0.002 | 0.01 | 6553 | 0.73 | -0.03 | 0.01 | 6554 | 0.01 | 0.03 | 0.01 | 6553 | 0.004 |
| FFMQ acting with awareness | 0.04 | 0.01 | 6554 | 0.004 | 0.004 | 0.01 | 6553 | 0.57 | -0.05 | 0.01 | 6554 | <.0001 | 0.08 | 0.01 | 6553 | <.0001 |
| FFMQ non-judging | 0.02 | 0.01 | 6554 | 0.04 | 0.003 | 0.01 | 6553 | 0.64 | -0.05 | 0.01 | 6554 | <.0001 | 0.04 | 0.01 | 6553 | <.0001 |
| FFMQ non-reactivity | 0.03 | 0.01 | 6554 | 0.04 | 0.01 | 0.01 | 6553 | 0.17 | -0.06 | 0.01 | 6554 | <.0001 | 0.06 | 0.01 | 6553 | <.0001 |
| MAAS | -0.13 | 0.08 | 6554 | 0.09 | -0.01 | 0.05 | 6553 | 0.80 | 0.42 | 0.07 | 6554 | <.0001 | -0.54 | 0.06 | 6553 | <.0001 |
| SOC elective selection | 0.01 | 0.08 | 6554 | 0.93 | -0.04 | 0.05 | 6553 | 0.50 | 0.04 | 0.08 | 6554 | 0.59 | 0.16 | 0.08 | 6553 | 0.03 |
| SOC loss-based selection | 0.13 | 0.07 | 6554 | 0.07 | -0.03 | 0.05 | 6553 | 0.50 | -0.02 | 0.07 | 6554 | 0.80 | 0.11 | 0.07 | 6553 | 0.12 |
| SOC optimization | 0.14 | 0.06 | 6554 | 0.01 | 0.07 | 0.04 | 6553 | 0.08 | -0.15 | 0.06 | 6554 | 0.01 | 0.20 | 0.05 | 6553 | <.001 |
| SOC compensation | 0.11 | 0.07 | 6554 | 0.11 | 0.06 | 0.05 | 6553 | 0.15 | -0.23 | 0.06 | 6554 | <.001 | 0.21 | 0.06 | 6553 | <.001 |
| UPPS-P lack of premeditation | -0.14 | 0.13 | 6554 | 0.28 | 0.27 | 0.09 | 6553 | 0.002 | 0.05 | 0.13 | 6554 | 0.72 | -0.26 | 0.13 | 6553 | 0.04 |
| UPPS-P lack of perseverance | -0.53 | 0.11 | 6554 | <.0001 | -0.15 | 0.08 | 6553 | 0.0498 | 0.42 | 0.11 | 6554 | <.001 | -0.47 | 0.10 | 6553 | <.0001 |
| Eysenck I-7 venturesomeness | 0.003 | 0.02 | 6554 | 0.84 | 0.07 | 0.01 | 6553 | <.0001 | -0.03 | 0.02 | 6554 | 0.09 | 0.03 | 0.02 | 6553 | 0.07 |

Supplemental Table 5. Baseline measures, association with momentary self-regulation in the **smoking sample**. Parameter estimates from mixed-effects models.

|  | **Momentary Perseverance** | | | | **Momentary Sensation Seeking** | | | | **Momentary Self-Judgment** | | | | **Momentary Mindfulness** | | | |
| --- | --- | --- | --- | --- | --- | --- | --- | --- | --- | --- | --- | --- | --- | --- | --- | --- |
| **Baseline measure** | β | SE | DF | p-value | β | SE | DF | p-value | β | SE | DF | p-value | β | SE | DF | p-value |
| RED-13 | -0.02 | 0.01 | 3150 | 0.09 | -0.01 | 0.01 | 3150 | 0.36 | 0.03 | 0.01 | 3150 | 0.002 | -0.04 | 0.01 | 3149 | <.0001 |
| SSRQ | 0.02 | 0.01 | 3150 | <.001 | 0.01 | 0.004 | 3150 | 0.06 | -0.01 | 0.005 | 3150 | 0.004 | 0.02 | 0.004 | 3149 | <.0001 |
| ERQ cognitive reappraisal | 0.03 | 0.01 | 3150 | 0.0514 | 0.01 | 0.01 | 3150 | 0.59 | -0.03 | 0.01 | 3150 | 0.02 | 0.03 | 0.01 | 3149 | 0.02 |
| ERQ expressive suppression | -0.01 | 0.02 | 3150 | 0.55 | 0.001 | 0.01 | 3150 | 0.96 | 0.03 | 0.02 | 3150 | 0.02 | -0.01 | 0.02 | 3149 | 0.56 |
| FFMQ observing | 0.06 | 0.02 | 3150 | 0.002 | 0.02 | 0.01 | 3150 | 0.23 | -0.005 | 0.02 | 3150 | 0.79 | 0.03 | 0.02 | 3149 | 0.08 |
| FFMQ describing | 0.03 | 0.01 | 3150 | 0.02 | 0.0003 | 0.01 | 3150 | 0.98 | -0.01 | 0.01 | 3150 | 0.28 | 0.02 | 0.01 | 3149 | 0.10 |
| FFMQ acting with awareness | 0.04 | 0.02 | 3150 | 0.03 | 0.01 | 0.01 | 3150 | 0.18 | -0.05 | 0.01 | 3150 | <.001 | 0.05 | 0.01 | 3149 | <.0001 |
| FFMQ non-judging | 0.01 | 0.01 | 3150 | 0.33 | 0.005 | 0.01 | 3150 | 0.59 | -0.05 | 0.01 | 3150 | <.0001 | 0.03 | 0.01 | 3149 | 0.003 |
| FFMQ non-reactivity | 0.04 | 0.02 | 3150 | 0.09 | 0.01 | 0.02 | 3150 | 0.42 | -0.06 | 0.02 | 3150 | 0.003 | 0.06 | 0.02 | 3149 | 0.002 |
| MAAS | -0.11 | 0.12 | 3150 | 0.36 | -0.07 | 0.07 | 3150 | 0.31 | 0.38 | 0.09 | 3150 | <.0001 | -0.42 | 0.08 | 3149 | <.0001 |
| SOC elective selection | 0.01 | 0.13 | 3150 | 0.94 | 0.01 | 0.08 | 3150 | 0.86 | 0.03 | 0.10 | 3150 | 0.78 | 0.16 | 0.10 | 3149 | 0.11 |
| SOC loss-based selection | 0.18 | 0.12 | 3150 | 0.11 | -0.06 | 0.07 | 3150 | 0.39 | -0.02 | 0.10 | 3150 | 0.83 | 0.11 | 0.09 | 3149 | 0.22 |
| SOC optimization | 0.21 | 0.09 | 3150 | 0.02 | 0.11 | 0.06 | 3150 | 0.06 | -0.12 | 0.07 | 3150 | 0.12 | 0.22 | 0.07 | 3149 | 0.001 |
| SOC compensation | 0.24 | 0.10 | 3150 | 0.02 | 0.09 | 0.06 | 3150 | 0.16 | -0.22 | 0.08 | 3150 | 0.01 | 0.22 | 0.08 | 3149 | 0.01 |
| UPPS-P lack of premeditation | -0.23 | 0.21 | 3150 | 0.28 | 0.07 | 0.13 | 3150 | 0.61 | 0.19 | 0.17 | 3150 | 0.27 | -0.44 | 0.16 | 3149 | 0.01 |
| UPPS-P lack of perseverance | -0.62 | 0.16 | 3150 | <.0001 | -0.25 | 0.10 | 3150 | 0.01 | 0.39 | 0.13 | 3150 | 0.004 | -0.44 | 0.13 | 3149 | <.001 |
| Eysenck I-7 venturesomeness | 0.01 | 0.02 | 3150 | 0.53 | 0.06 | 0.01 | 3150 | <.0001 | 0.02 | 0.02 | 3150 | 0.22 | 0.01 | 0.02 | 3149 | 0.44 |

Supplemental Table 6. Baseline measures, association with momentary self-regulation in the **binge-eating sample**. Parameter estimates from mixed-effects models.

|  | **Momentary Perseverance** | | | | **Momentary Sensation Seeking** | | | | **Momentary Self-Judgment** | | | | **Momentary Mindfulness** | | | |
| --- | --- | --- | --- | --- | --- | --- | --- | --- | --- | --- | --- | --- | --- | --- | --- | --- |
| **Baseline measure** | β | SE | DF | p-value | β | SE | DF | p-value | β | SE | DF | p-value | β | SE | DF | p-value |
| RED-13 | -0.01 | 0.02 | 3403 | 0.76 | -0.03 | 0.01 | 3402 | 0.01 | 0.02 | 0.02 | 3403 | 0.25 | -0.02 | 0.02 | 3403 | 0.18 |
| SSRQ | 0.02 | 0.01 | 3403 | 0.001 | 0.01 | 0.005 | 3402 | 0.07 | -0.01 | 0.01 | 3403 | 0.04 | 0.02 | 0.01 | 3403 | 0.01 |
| ERQ cognitive reappraisal | 0.03 | 0.01 | 3403 | 0.01 | 0.03 | 0.01 | 3402 | 0.01 | -0.02 | 0.01 | 3403 | 0.21 | 0.01 | 0.01 | 3403 | 0.60 |
| ERQ expressive suppression | -0.02 | 0.02 | 3403 | 0.22 | 0.01 | 0.01 | 3402 | 0.54 | 0.02 | 0.02 | 3403 | 0.32 | -0.01 | 0.02 | 3403 | 0.51 |
| FFMQ observing | 0.01 | 0.02 | 3403 | 0.45 | 0.002 | 0.01 | 3402 | 0.90 | -0.02 | 0.02 | 3403 | 0.18 | 0.03 | 0.02 | 3403 | 0.07 |
| FFMQ describing | 0.05 | 0.01 | 3403 | <.001 | 0.004 | 0.01 | 3402 | 0.68 | -0.04 | 0.01 | 3403 | 0.002 | 0.04 | 0.01 | 3403 | 0.01 |
| FFMQ acting with awareness | 0.06 | 0.02 | 3403 | <.0001 | -0.01 | 0.01 | 3402 | 0.59 | -0.03 | 0.02 | 3403 | 0.04 | 0.09 | 0.01 | 3403 | <.0001 |
| FFMQ non-judging | 0.04 | 0.01 | 3403 | 0.003 | 0.008 | 0.01 | 3402 | 0.93 | -0.04 | 0.01 | 3403 | <.001 | 0.02 | 0.01 | 3403 | 0.08 |
| FFMQ non-reactivity | 0.04 | 0.02 | 3403 | 0.04 | 0.02 | 0.01 | 3402 | 0.24 | -0.03 | 0.02 | 3403 | 0.18 | 0.03 | 0.02 | 3403 | 0.14 |
| MAAS | -0.35 | 0.12 | 3403 | 0.003 | 0.07 | 0.09 | 3402 | 0.43 | 0.35 | 0.12 | 3403 | 0.004 | -0.58 | 0.10 | 3403 | <.0001 |
| SOC elective selection | 0.01 | 0.10 | 3403 | 0.95 | -0.08 | 0.08 | 3402 | 0.28 | 0.06 | 0.11 | 3403 | 0.58 | 0.16 | 0.10 | 3403 | 0.12 |
| SOC loss-based selection | 0.09 | 0.09 | 3403 | 0.30 | -0.01 | 0.07 | 3402 | 0.88 | -0.004 | 0.10 | 3403 | 0.96 | 0.09 | 0.09 | 3403 | 0.32 |
| SOC optimization | 0.14 | 0.08 | 3403 | 0.06 | 0.04 | 0.06 | 3402 | 0.46 | -0.08 | 0.08 | 3403 | 0.36 | 0.07 | 0.08 | 3403 | 0.40 |
| SOC compensation | 0.02 | 0.09 | 3403 | 0.85 | 0.04 | 0.07 | 3402 | 0.52 | -0.14 | 0.09 | 3403 | 0.13 | 0.10 | 0.09 | 3403 | 0.29 |
| UPPS-P lack of premeditation | -0.05 | 0.17 | 3403 | 0.77 | 0.45 | 0.11 | 3402 | <.0001 | 0.04 | 0.18 | 3403 | 0.82 | -0.22 | 0.17 | 3403 | 0.20 |
| UPPS-P lack of perseverance | -0.53 | 0.15 | 3403 | <.001 | -0.04 | 0.12 | 3402 | 0.71 | 0.28 | 0.17 | 3403 | 0.10 | -0.33 | 0.16 | 3403 | 0.04 |
| Eysenck I-7 venturesomeness | -0.002 | 0.02 | 3403 | 0.92 | 0.08 | 0.01 | 3402 | <.0001 | -0.06 | 0.02 | 3403 | 0.01 | 0.02 | 0.02 | 3403 | 0.47 |

Supplemental Table 7. Momentary context measures, association with momentary self-regulation in the **smoking sample**. Parameter estimates from mixed-effects models.

|  | **Momentary Perseverance** | | | | **Momentary Sensation Seeking** | | | | **Momentary Self-Judgment** | | | | **Momentary Mindfulness** | | | |
| --- | --- | --- | --- | --- | --- | --- | --- | --- | --- | --- | --- | --- | --- | --- | --- | --- |
| **Momentary context** | β | SE | DF | p-value | β | SE | DF | p-value | β | SE | DF | p-value | β | SE | DF | p-value |
| Alone | -0.11 | 0.03 | 3149 | 0.002 | -0.01 | 0.02 | 3149 | 0.46 | 0.005 | 0.02 | 3149 | 0.83 | 0.01 | 0.03 | 3148 | 0.68 |
| At this moment, location: |  |  |  |  |  |  |  |  |  |  |  |  |  |  |  |  |
| Car | 0.20 | 0.05 | 3147 | <.001 | -0.01 | 0.03 | 3147 | 0.68 | -0.04 | 0.04 | 3147 | 0.23 | -0.06 | 0.04 | 3146 | 0.18 |
| Other | 0.08 | 0.06 | 3147 | 0.24 | 0.07 | 0.04 | 3147 | 0.05 | -0.07 | 0.05 | 3147 | 0.10 | 0.02 | 0.05 | 3146 | 0.66 |
| Work | 0.35 | 0.04 | 3147 | <.0001 | 0.02 | 0.02 | 3147 | 0.36 | -0.05 | 0.03 | 3147 | 0.07 | -0.17 | 0.03 | 3146 | <.0001 |
| Home (reference) | 0.00 |  |  |  | 0.00 |  |  |  | 0.00 |  |  |  | 0.00 |  |  |  |
| Right now: positive affect | 0.20 | 0.02 | 3149 | <.0001 | 0.04 | 0.01 | 3149 | <.0001 | -0.10 | 0.01 | 3149 | <.0001 | 0.09 | 0.01 | 3148 | <.0001 |
| Right now: negative affect | -0.13 | 0.02 | 3149 | <.0001 | -0.01 | 0.01 | 3149 | 0.28 | 0.17 | 0.01 | 3149 | <.0001 | -0.10 | 0.01 | 3148 | <.0001 |
| Right now: how stressed? | -0.05 | 0.01 | 3149 | <.0001 | -0.02 | 0.01 | 3149 | 0.004 | 0.08 | 0.01 | 3149 | <.0001 | -0.05 | 0.01 | 3148 | <.0001 |
| Right now: how tired? | -0.05 | 0.01 | 3149 | <.0001 | -0.001 | 0.01 | 3149 | 0.84 | 0.03 | 0.01 | 3149 | <.0001 | -0.04 | 0.01 | 3148 | <.0001 |
| Used Laddr (≤12 hrs) | 0.01 | 0.04 | 3149 | 0.76 | -0.02 | 0.02 | 3149 | 0.29 | 0.02 | 0.02 | 3149 | 0.53 | -0.04 | 0.03 | 3148 | 0.15 |
| Used Laddr (≤1 hr) | -0.04 | 0.05 | 3149 | 0.46 | -0.03 | 0.03 | 3149 | 0.22 | 0.01 | 0.03 | 3149 | 0.81 | -0.05 | 0.04 | 3148 | 0.22 |

Supplemental Table 8. Momentary context measures, association with momentary self-regulation by recent Laddr use (≤12 hrs) in the **smoking sample**. Parameter estimates from mixed-effects models with a context-by-Laddr interaction term.

|  | **Momentary Perseverance** | | | | **Momentary Sensation Seeking** | | | | **Momentary Self-Judgment** | | | | **Momentary Mindfulness** | | | |
| --- | --- | --- | --- | --- | --- | --- | --- | --- | --- | --- | --- | --- | --- | --- | --- | --- |
| **Association between momentary context and momentary self-regulation measures**  **Presented separately by recent Laddr use** (≤12 hrs) | β | SE | DF | p-value | β | SE | DF | p-value | β | SE | DF | p-value | β | SE | DF | p-value |
| **Momentary context** |  |  |  |  |  |  |  |  |  |  |  |  |  |  |  |  |
| Alone |  |  |  | 0.82 |  |  |  | 0.81 |  |  |  | 0.19 |  |  |  | 0.35 |
| Laddr use (≤12 hrs) | -0.10 | 0.05 | 3148 | 0.04 | -0.02 | 0.03 | 3148 | 0.38 | -0.03 | 0.03 | 3148 | 0.39 | 0.04 | 0.04 | 3147 | 0.35 |
| No Laddr (≤12 hrs) | -0.08 | 0.04 | 3148 | 0.05 | -0.02 | 0.02 | 3148 | 0.52 | 0.03 | 0.03 | 3148 | 0.36 | -0.01 | 0.03 | 3147 | 0.78 |
| At this moment, location: |  |  |  |  |  |  |  |  |  |  |  |  |  |  |  |  |
| Car |  |  |  | 0.66 |  |  |  | 0.68 |  |  |  | 0.81 |  |  |  | 0.29 |
| Laddr use (≤12 hrs) | 0.16 | 0.08 | 3144 | 0.05 | -0.02 | 0.05 | 3144 | 0.61 | -0.05 | 0.06 | 3144 | 0.34 | -0.11 | 0.06 | 3143 | 0.09 |
| No Laddr (≤12 hrs) | 0.21 | 0.07 | 3144 | 0.003 | 0.001 | 0.04 | 3144 | 0.97 | -0.04 | 0.05 | 3144 | 0.46 | -0.02 | 0.05 | 3143 | 0.70 |
| Other |  |  |  | 0.98 |  |  |  | 0.28 |  |  |  | 0.18 |  |  |  | 0.73 |
| Laddr use (≤12 hrs) | 0.08 | 0.09 | 3144 | 0.35 | 0.11 | 0.05 | 3144 | 0.04 | -0.02 | 0.06 | 3144 | 0.77 | 0.01 | 0.07 | 3143 | 0.91 |
| No Laddr (≤12 hrs) | 0.09 | 0.09 | 3144 | 0.34 | 0.03 | 0.05 | 3144 | 0.57 | -0.14 | 0.06 | 3144 | 0.03 | 0.04 | 0.07 | 3143 | 0.56 |
| Work |  |  |  | 0.66 |  |  |  | 0.02 |  |  |  | 0.52 |  |  |  | 0.53 |
| Laddr use (≤12 hrs) | 0.37 | 0.06 | 3144 | <.0001 | -0.04 | 0.04 | 3144 | 0.24 | -0.03 | 0.04 | 3144 | 0.45 | -0.19 | 0.05 | 3143 | <.001 |
| No Laddr (≤12 hrs) | 0.34 | 0.05 | 3144 | <.0001 | 0.07 | 0.03 | 3144 | 0.03 | -0.07 | 0.04 | 3144 | 0.07 | -0.15 | 0.04 | 3143 | <.001 |
| Home (reference) | 0.00 |  |  |  | 0.00 |  |  |  | 0.00 |  |  |  | 0.00 |  |  |  |
| Right now: positive affect |  |  |  | 0.40 |  |  |  | 0.005 |  |  |  | 0.58 |  |  |  | 0.53 |
| Laddr use (≤12 hrs) | 0.17 | 0.02 | 3148 | <.0001 | 0.06 | 0.01 | 3148 | <.0001 | -0.10 | 0.01 | 3148 | <.0001 | 0.09 | 0.02 | 3147 | <.0001 |
| No Laddr (≤12 hrs) | 0.19 | 0.02 | 3148 | <.0001 | 0.02 | 0.01 | 3148 | 0.04 | -0.09 | 0.01 | 3148 | <.0001 | 0.08 | 0.01 | 3147 | <.0001 |
| Right now: negative affect |  |  |  | 0.71 |  |  |  | 0.99 |  |  |  | 0.16 |  |  |  | 0.62 |
| Laddr use (≤12 hrs) | -0.14 | 0.02 | 3148 | <.0001 | -0.01 | 0.01 | 3148 | 0.59 | 0.18 | 0.01 | 3148 | <.0001 | -0.10 | 0.02 | 3147 | <.0001 |
| No Laddr (≤12 hrs) | -0.14 | 0.02 | 3148 | <.0001 | -0.01 | 0.01 | 3148 | 0.56 | 0.16 | 0.01 | 3148 | <.0001 | -0.09 | 0.02 | 3147 | <.0001 |
| Right now: how stressed? |  |  |  | 0.40 |  |  |  | 0.17 |  |  |  | 0.14 |  |  |  | 0.48 |
| Laddr use (≤12 hrs) | -0.04 | 0.01 | 3148 | 0.002 | -0.02 | 0.01 | 3148 | 0.004 | 0.09 | 0.01 | 3148 | <.0001 | -0.06 | 0.01 | 3147 | <.0001 |
| No Laddr (≤12 hrs) | -0.06 | 0.01 | 3148 | <.0001 | -0.01 | 0.01 | 3148 | 0.19 | 0.07 | 0.01 | 3148 | <.0001 | -0.05 | 0.01 | 3147 | <.0001 |
| Right now: how tired? |  |  |  | 0.59 |  |  |  | 0.10 |  |  |  | 0.87 |  |  |  | 0.34 |
| Laddr use (≤12 hrs) | -0.06 | 0.01 | 3148 | <.0001 | -0.01 | 0.01 | 3148 | 0.23 | 0.03 | 0.01 | 3148 | 0.002 | -0.05 | 0.01 | 3147 | <.0001 |
| No Laddr (≤12 hrs) | -0.05 | 0.01 | 3148 | <.001 | 0.01 | 0.01 | 3148 | 0.35 | 0.03 | 0.01 | 3148 | <.001 | -0.04 | 0.01 | 3147 | <.001 |

Supplemental Table 9. Momentary context measures, association with momentary self-regulation in the **binge-eating sample**. Parameter estimates from mixed-effects models.

|  | **Momentary Perseverance** | | | | **Momentary Sensation Seeking** | | | | **Momentary Self-Judgment** | | | | **Momentary Mindfulness** | | | |
| --- | --- | --- | --- | --- | --- | --- | --- | --- | --- | --- | --- | --- | --- | --- | --- | --- |
| **Momentary context** | β | SE | DF | p-value | β | SE | DF | p-value | β | SE | DF | p-value | β | SE | DF | p-value |
| Alone | -0.03 | 0.03 | 3402 | 0.43 | -0.09 | 0.02 | 3401 | <.0001 | 0.01 | 0.03 | 3402 | 0.65 | -0.04 | 0.03 | 3402 | 0.14 |
| At this moment, location: |  |  |  |  |  |  |  |  |  |  |  |  |  |  |  |  |
| Car | 0.23 | 0.05 | 3400 | <.0001 | 0.03 | 0.03 | 3399 | 0.39 | 0.01 | 0.05 | 3400 | 0.81 | -0.05 | 0.05 | 3400 | 0.26 |
| Other | 0.03 | 0.04 | 3400 | 0.52 | 0.13 | 0.03 | 3399 | <.0001 | -0.08 | 0.04 | 3400 | 0.03 | 0.10 | 0.04 | 3400 | 0.01 |
| Work | 0.21 | 0.04 | 3400 | <.0001 | -0.02 | 0.02 | 3399 | 0.49 | -0.10 | 0.03 | 3400 | 0.004 | -0.16 | 0.03 | 3400 | <.0001 |
| Home (reference) | 0.00 |  |  |  | 0.00 |  |  |  | 0.00 |  |  |  | 0.00 |  |  |  |
| Right now: positive affect | 0.21 | 0.01 | 3402 | <.0001 | 0.07 | 0.01 | 3401 | <.0001 | -0.16 | 0.01 | 3402 | <.0001 | 0.17 | 0.01 | 3402 | <.0001 |
| Right now: negative affect | -0.14 | 0.01 | 3402 | <.0001 | -0.04 | 0.01 | 3401 | <.0001 | 0.21 | 0.01 | 3402 | <.0001 | -0.19 | 0.01 | 3402 | <.0001 |
| Right now: how stressed? | -0.05 | 0.01 | 3402 | <.0001 | -0.03 | 0.01 | 3401 | <.0001 | 0.10 | 0.01 | 3402 | <.0001 | -0.11 | 0.01 | 3402 | <.0001 |
| Right now: how tired? | -0.08 | 0.01 | 3402 | <.0001 | -0.01 | 0.01 | 3401 | 0.30 | 0.04 | 0.01 | 3402 | <.0001 | -0.08 | 0.01 | 3402 | <.0001 |
| Used Laddr (≤12 hrs) | 0.01 | 0.03 | 3402 | 0.76 | 0.01 | 0.02 | 3401 | 0.57 | -0.01 | 0.03 | 3402 | 0.79 | -0.03 | 0.03 | 3402 | 0.36 |
| Used Laddr (≤1 hr) | -0.01 | 0.05 | 3402 | 0.88 | 0.05 | 0.03 | 3401 | 0.12 | 0.08 | 0.04 | 3402 | 0.06 | -0.05 | 0.04 | 3402 | 0.26 |

Supplemental Table 10. Momentary context measures, association with momentary self-regulation by recent Laddr use (≤12 hrs) in the **binge-eating sample**. Parameter estimates from mixed-effects models with a context-by-Laddr interaction term.

|  | **Momentary Perseverance** | | | | **Momentary Sensation Seeking** | | | | **Momentary Self-Judgment** | | | | **Momentary Mindfulness** | | | |
| --- | --- | --- | --- | --- | --- | --- | --- | --- | --- | --- | --- | --- | --- | --- | --- | --- |
| **Association between momentary context and momentary self-regulation measures**  **Presented separately by recent Laddr use** (≤12 hrs) | β | SE | DF | p-value | β | SE | DF | p-value | β | SE | DF | p-value | β | SE | DF | p-value |
| **Momentary context** |  |  |  |  |  |  |  |  |  |  |  |  |  |  |  |  |
| Alone |  |  |  | 0.94 |  |  |  | 0.54 |  |  |  | 0.67 |  |  |  | 0.74 |
| Laddr use (≤12 hrs) | -0.01 | 0.04 | 3401 | 0.76 | -0.08 | 0.03 | 3400 | 0.002 | 0.03 | 0.04 | 3401 | 0.50 | -0.05 | 0.04 | 3401 | 0.21 |
| No Laddr (≤12 hrs) | -0.02 | 0.04 | 3401 | 0.69 | -0.11 | 0.03 | 3400 | <.001 | 0.003 | 0.04 | 3401 | 0.93 | -0.03 | 0.04 | 3401 | 0.44 |
| At this moment, location: |  |  |  |  |  |  |  |  |  |  |  |  |  |  |  |  |
| Car |  |  |  | 0.91 |  |  |  | 0.30 |  |  |  | 0.76 |  |  |  | 0.59 |
| Laddr use (≤12 hrs) | 0.24 | 0.08 | 3397 | 0.001 | -0.01 | 0.05 | 3396 | 0.90 | -0.002 | 0.07 | 3397 | 0.98 | -0.08 | 0.07 | 3397 | 0.25 |
| No Laddr (≤12 hrs) | 0.23 | 0.08 | 3397 | 0.003 | 0.06 | 0.05 | 3396 | 0.19 | 0.03 | 0.07 | 3397 | 0.68 | -0.02 | 0.07 | 3397 | 0.72 |
| Other |  |  |  | 0.60 |  |  |  | 0.37 |  |  |  | 0.98 |  |  |  | 0.18 |
| Laddr use (≤12 hrs) | 0.05 | 0.06 | 3397 | 0.39 | 0.15 | 0.04 | 3396 | <.0001 | -0.08 | 0.05 | 3397 | 0.10 | 0.06 | 0.05 | 3397 | 0.29 |
| No Laddr (≤12 hrs) | 0.01 | 0.06 | 3397 | 0.91 | 0.10 | 0.04 | 3396 | 0.01 | -0.08 | 0.05 | 3397 | 0.12 | 0.16 | 0.05 | 3397 | 0.005 |
| Work |  |  |  | 0.01 |  |  |  | 0.64 |  |  |  | 0.69 |  |  |  | 0.04 |
| Laddr use (≤12 hrs) | 0.12 | 0.05 | 3397 | 0.03 | -0.03 | 0.03 | 3396 | 0.42 | -0.11 | 0.05 | 3397 | 0.02 | -0.23 | 0.05 | 3397 | <.0001 |
| No Laddr (≤12 hrs) | 0.30 | 0.05 | 3397 | <.0001 | -0.01 | 0.03 | 3396 | 0.88 | -0.09 | 0.05 | 3397 | 0.07 | -0.09 | 0.05 | 3397 | 0.06 |
| Home (reference) | 0.00 |  |  |  | 0.00 |  |  |  | 0.00 |  |  |  | 0.00 |  |  |  |
| Right now: positive affect |  |  |  | 0.92 |  |  |  | 0.75 |  |  |  | 0.13 |  |  |  | 0.39 |
| Laddr use (≤12 hrs) | 0.21 | 0.02 | 3401 | <.0001 | 0.07 | 0.01 | 3400 | <.0001 | -0.18 | 0.02 | 3401 | <.0001 | 0.18 | 0.02 | 3401 | <.0001 |
| No Laddr (≤12 hrs) | 0.21 | 0.02 | 3401 | <.0001 | 0.07 | 0.01 | 3400 | <.0001 | -0.15 | 0.02 | 3401 | <.0001 | 0.16 | 0.02 | 3401 | <.0001 |
| Right now: negative affect |  |  |  | 0.13 |  |  |  | 0.33 |  |  |  | 0.34 |  |  |  | 0.14 |
| Laddr use (≤12 hrs) | -0.17 | 0.02 | 3401 | <.0001 | -0.05 | 0.01 | 3400 | <.0001 | 0.20 | 0.02 | 3401 | <.0001 | -0.20 | 0.02 | 3401 | <.0001 |
| No Laddr (≤12 hrs) | -0.13 | 0.02 | 3401 | <.0001 | -0.03 | 0.01 | 3400 | 0.01 | 0.22 | 0.02 | 3401 | <.0001 | -0.17 | 0.02 | 3401 | <.0001 |
| Right now: how stressed? |  |  |  | 0.29 |  |  |  | 0.64 |  |  |  | 0.65 |  |  |  | 0.08 |
| Laddr use (≤12 hrs) | -0.07 | 0.01 | 3401 | <.0001 | -0.03 | 0.01 | 3400 | <.001 | 0.10 | 0.01 | 3401 | <.0001 | -0.13 | 0.01 | 3401 | <.0001 |
| No Laddr (≤12 hrs) | -0.05 | 0.01 | 3401 | <.001 | -0.03 | 0.01 | 3400 | 0.003 | 0.09 | 0.01 | 3401 | <.0001 | -0.10 | 0.01 | 3401 | <.0001 |
| Right now: how tired? |  |  |  | 0.94 |  |  |  | 0.27 |  |  |  | 0.31 |  |  |  | 0.22 |
| Laddr use (≤12 hrs) | -0.08 | 0.01 | 3401 | <.0001 | -0.01 | 0.01 | 3400 | 0.13 | 0.05 | 0.01 | 3401 | <.0001 | -0.09 | 0.01 | 3401 | <.0001 |
| No Laddr (≤12 hrs) | -0.08 | 0.01 | 3401 | <.0001 | -0.001 | 0.01 | 3400 | 0.93 | 0.04 | 0.01 | 3401 | 0.002 | -0.07 | 0.01 | 3401 | <.0001 |
